# Clinical data mining reveals analgesic effects of lapatinib in cancer patients

**DOI:** 10.1038/s41598-021-82318-w

**Published:** 2021-02-11

**Authors:** Shuo Zhou, Fang Zheng, Chang-Guo Zhan

**Affiliations:** 1 Molecular Modeling and Biopharmaceutical Center, College of Pharmacy, University of Kentucky, 789 South Limestone Street, Lexington, KY 40536 USA; 2 Department of Pharmaceutical Sciences, College of Pharmacy, University of Kentucky, 789 South Limestone Street, Lexington, KY 40536 USA

**Keywords:** Computational biology and bioinformatics, Cancer

## Abstract

Microsomal prostaglandin E2 synthase 1 (mPGES-1) is recognized as a promising target for a next generation of anti-inflammatory drugs that are not expected to have the side effects of currently available anti-inflammatory drugs. Lapatinib, an FDA-approved drug for cancer treatment, has recently been identified as an mPGES-1 inhibitor. But the efficacy of lapatinib as an analgesic remains to be evaluated. In the present clinical data mining (CDM) study, we have collected and analyzed all lapatinib-related clinical data retrieved from clinicaltrials.gov. Our CDM utilized a meta-analysis protocol, but the clinical data analyzed were not limited to the primary and secondary outcomes of clinical trials, unlike conventional meta-analyses. All the pain-related data were used to determine the numbers and odd ratios (ORs) of various forms of pain in cancer patients with lapatinib treatment. The ORs, 95% confidence intervals, and *P* values for the differences in pain were calculated and the heterogeneous data across the trials were evaluated. For all forms of pain analyzed, the patients received lapatinib treatment have a reduced occurrence (OR 0.79; CI 0.70–0.89; *P* = 0.0002 for the overall effect). According to our CDM results, available clinical data for 12,765 patients enrolled in 20 randomized clinical trials indicate that lapatinib therapy is associated with a significant reduction in various forms of pain, including musculoskeletal pain, bone pain, headache, arthralgia, and pain in extremity, in cancer patients. Our CDM results have demonstrated the significant analgesic effects of lapatinib, suggesting that lapatinib may be repurposed as a novel type of analgesic.

## Introduction

Currently used non-steroid anti-inflammatory drugs (NSAIDs) target cyclooxygenase (COX)-1 and/or COX-2 with the goal to decrease biosynthesis of pro-inflammatory prostaglandin E2 (PGE_2_) for pain relief. This is because, in general, pain starts from tissue damage/injury and the corresponding inflammation response^1^. So, excessive inflammation is the root etiology of pain. NSAIDs are still the most accessible analgesics in clinical market^2,3^. However, all the COX-1/2 inhibitors have undesirable adverse effects, including significant cardiovascular, cerebrovascular, and cardiovascular risks^4,5^, because the COX-1/2 inhibition also blocks normal production of other physiologically necessary prostaglandins. As an inducible enzyme, microsomal prostaglandin E2 synthase 1 (mPGES-1), which exists in the downstream of the COX enzymes^6^, is a more promising, ideal target for anti-inflammatory pain relief. This is because the mPGES-1 inhibition will directly and only block the inducible over-production of PGE_2_ without blocking the normal production of other prostaglandins and basal level of PGE_2_ for physiological homeostasis, as confirmed in extensive studies using mPGES-1 knockouts^7–15^. Hence, a highly selective mPGES-1 inhibitor is expected to retain the anti-inflammatory and analgesic effects of COX-1/2 inhibitors, but without the COX-1/2 inhibition-caused side effects. To develop an improved anti-inflammatory drug, various mPGES-1 inhibitors have been reported in the literature^16–37^. Unfortunately, few of the previously reported potent inhibitors of human mPGES-1 have been demonstrated to also be potent inhibitors of mouse or rat mPGES-1, preventing the use of well-established mouse/rat models of pain or other inflammation-related diseases for preclinical studies^38^.

To develop a next generation of anti-inflammatory drug targeting mPGES-1 for pain relief, we recently modeled and validated human mPGES-1 structure in an open conformation showing a more conserved glutathione (GSH)-binding site^39^. The open-conformation structure was then used to virtually screen FDA-approved drugs for their potential inhibitory activity against human mPGES-1^40^. Following the virtual screening, in vitro activity assays revealed that lapatinib, approved by FDA to treat HER2-positive metastatic breast cancer in women who have already had chemotherapy and trastuzumab (Herceptin), can potently inhibit human mPGES-1 (IC_50_ = 0.8 µM) and less potently inhibit mouse mPGES-1 (IC_50_ = 12 µM)^40^. Even if lapatinib has a 15-fold lower inhibitory activity against mouse mPGES-1 compared to human mPGES-1, oral administration (PO) of lapatinib significantly and dose-dependently decreased the air-pouch PGE_2_ level in carrageenan-induced mouse model of inflammation, demonstrating the desirable anti-inflammatory effects^40^. These data suggest that lapatinib should have even stronger anti-inflammatory effects in humans. Further analysis^40^ of available clinical pharmacokinetic (PK) data revealed that the maximum drug concentration (C_max_) values of lapatinib from five different clinical trials are much higher than the IC_50_ in humans (e.g. C_max_ = 8.598 µM under the FDA-approved oral dose of 1500 mg once a day), suggesting that lapatinib might have significant anti-inflammatory and analgesic effects even with an oral dose a few times lower than 1500 mg.

Notably, there have been hundreds of lapatinib-involved clinical trials (including 327 trials registered in clinicaltrails.gov) so far. However, none of the trials has had pain relief listed as one of the primary or secondary outcomes. To the best of our knowledge, only one of the trials was designed to investigate the impact of lapatinib monotherapy on the quality of life (QoL) and pain symptoms in patients with HER2+ relapsed or refractory inflammatory breast cancer^41^. Specifically, a Phase II trial concluded that lapatinib monotherapy may “provide relief from symptoms, including pain, in the short term”^41^. However, their sample size was too small (n = 17 for the lapatinib group) to achieve the statistical significance in terms of the analgesic effects of lapatinib. There has been no report of any relevant meta-analysis concerning analgesic effects of lapatinib. This is because, in general, conventional methods of meta-analysis have been focused on analysis of available clinical data listed as the primary or secondary outcomes of clinical trials, and the required primary or secondary outcomes of clinical trials are not available for pain relief with lapatinib.

Nevertheless, as well known, many cancer patients suffer from pain. For this reason, we may reasonably assume that the pain information for the cancer patients could have been buried in other sections (*e.g*. adverse events) other than the primary and secondary outcomes in the released clinical trial records. So, in order to examine the possible analgesic effects of lapatinib, we decided to conduct a more detailed clinical data mining (CDM) study beyond the conventional meta-analysis. Specifically, we first collected all available lapatinib-related clinical data about pain beyond the primary and secondary outcomes of clinical trials, and then analyzed the collected clinical data by using a meta-analysis protocol. In other words, our meta-analysis was not limited to the primary and secondary outcomes of clinical trials such that more clinical data were available for our CDM or non-conventional meta-analysis. According to our CDM results, lapatinib therapy is associated with a significant reduction in various forms of pain, including musculoskeletal pain, bone pain, headache, arthralgia, and pain in extremity, in cancer patients. Our CDM data have clearly demonstrated the favorable analgesic effects of lapatinib.

## Results

### Search results and study quality

As described in the “Methods” section, all lapatinib-related clinical trial data about pain were retrieved from the MEDLINE database through clinicaltrails.gov, and the clinical data collected for analysis were not limited to the primary or secondary outcomes of the clinical trials. Within the clinical trials involving lapatinib, only randomized studies with a proper control group were selected for analysis. As a result, 20 randomized, controlled trials were identified based on the criteria mentioned in the Methods section, including a total of 12,765 cancer patients (see Table 1 and Supporting Information Tables S1 to 4 for the clinical data). In all these selected trials, four were quadruple-blind^42–45^, one was triple-blind^46^, five were double-blind^47–51^, one was single-blind^52^, and the other trials were not masked^53–61^. 14 trials recruited breast cancer patients^43–46,48,50,51,55–61^, three trials recruited head and neck cancer patients^47,49,52^, two trials recruited gastric cancer patients^42,53^, and one trial recruited cervical cancer patients^54^. Three studies were purely placebo-controlled^44,46,52^, three studies had a paclitaxel component^50,51,53^, five studies had a trastuzumab component^55–58,60^, two studies had a letrozole component^43,45^, two trials had a pazopanib component^48,54^, two trials had radiotherapy intervention^47,49^, one trial had both Paclitaxel and oxaliplatin components^42^, one trial had both paclitaxel and trastuzumab components^59^, and one trial had both trastuzumab and aromatase inhibitors (AI)^61^. All these trials were randomized with about equal numbers of individuals assigned to the lapatinib therapy and control groups, except for NCT00371566 which used a 2:1 ratio^52^ and NCT00558103 which used a 3:1 ratio^48^. Dosage of lapatinib ranged from 750 to 1500 mg daily. Summarized in Table S2 (Supporting Information) is analysis of the risks of bias about the clinical trials collected.

**Table 1:** Basic information about the clinical trials analyzed in the meta-analysis.

Clinical trial #	No. of individuals tolled	Male (%)	Patient type	Mean age (standard deviation) in years	Lapatinib dosage
NCT00486954^53^	272	79	Gastric cancer	60.5 (10.2)	1500 mg q.d
NCT00387127^47^	66	90	Head and neck cancer	56.1 (6.2)	1500 mg q.d
NCT00558103^48^	51	0	Breast cancer	53.0 (11.3)	1500 mg q.d
NCT00374322^46^	3147	0	Breast cancer	52.0 (9.9)	1500 mg q.d
NCT00430781^54^	150	0	Cervical cancer	49.5 (10.9)	1500 mg q.d
NCT00680901^42^	537	75	Gastric cancer	58.9 (11.2)	1500 mg q.d
NCT00553358^55^	301	0	Breast cancer	50.0 (23–80)*	1000 mg q.d
NCT00371566^52^	105	81	Head and neck cancer	57.1 (10.8)	1500 mg q.d
NCT00424255^49^	685	83	Head and neck cancer	53.8 (9.1)	1500 mg q.d
NCT00490139^56^	4137	0	Breast cancer	51.0 (10.2)	1000 mg q.d
NCT00073528^43^	1278	0	Breast cancer	63.1 (9.8)	1500 mg q.d
NCT00429299^57^	82	0	Breast cancer	49.3 (26–68)*	1000 mg q.d
NCT00390455^44^	278	0	Breast cancer	N/A	1500 mg q.d
NCT00422903^45^	92	0	Breast cancer	70 (47–88)*	1500 mg q.d
NCT00524303^58^	63	0	Breast cancer	50.4 (10.0)	1000 mg q.d
NCT00075270^50^	579	0	Breast cancer	51.8 (10.7)	1500 mg q.d
NCT00770809^59^	230	0	Breast cancer	49.4 (24–75)*	750 mg q.d
NCT00281658^51^	443	0	Breast cancer	49.2 (10.3)	1500 mg q.d
NCT00968968^60^	35	0	Breast cancer	56.5 (11.4)	1000 mg q.d
NCT01160211^61^	234	0	Breast cancer	56.4 (10.2)	1000 mg q.d

*The trials in which the age information was reported as mean (range). *N/A* not available.

### Major pain events

Among all investigated clinical trials, 23 different forms of pain were complained by the patients. Most of these forms of pain had a relatively small sample size when we combined the data from different trials, making the analyses on those forms of pain unreliable. Hence, only the forms of pain with more than 5000 patients involved in both the treatment and control groups are discussed here as the “major pain events”. A total of six forms of pain met these criteria, namely headache, bone pain, arthralgia, myalgia, pain in extremity, and musculoskeletal pain. Within the top-six, the patient count ranged from 6563 to 12,661. Outside of the top-six, the largest patient count was only 3438 (see Supporting Information Table S3).

**Figure 1:**
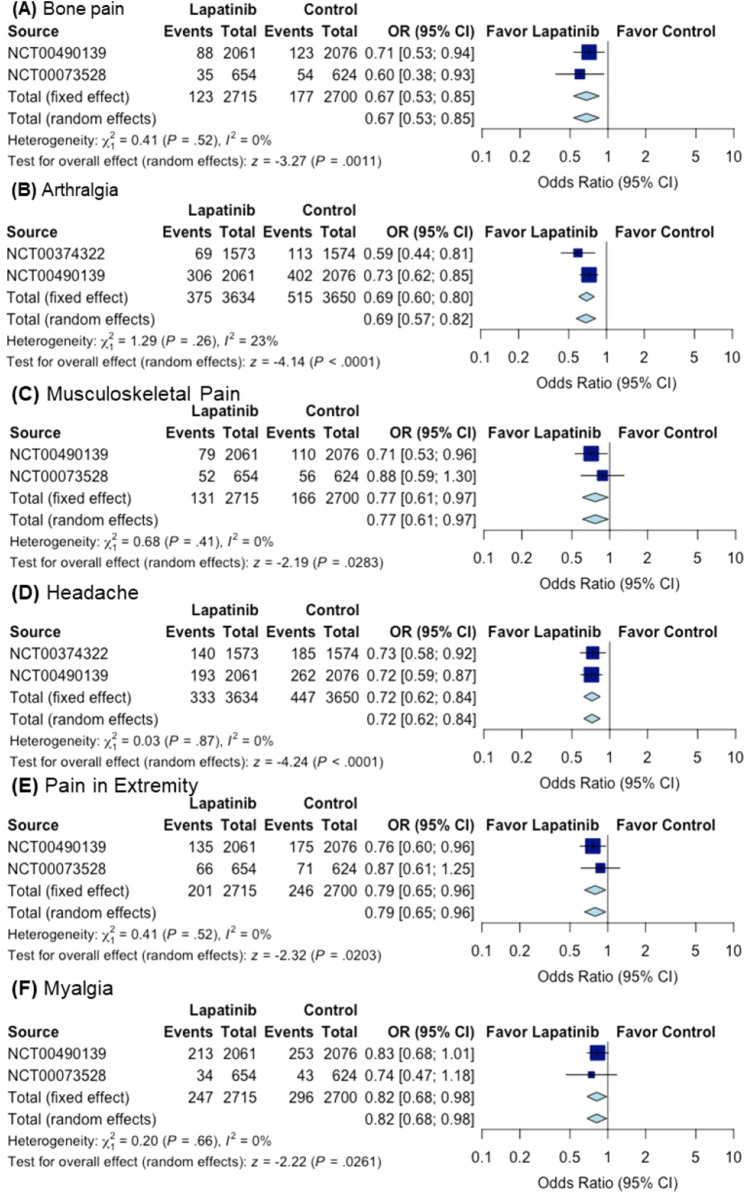
Overall effect of lapatinib treatment on (**A**) bone pain, (**B**) arthralgia, (**C**) musculoskeletal pain, (**D**) headache, (**E**) pain in extremity, and (**F**) myalgia in the largest two trials for each form of pain. The corresponding figures of the full meta-analysis with all the trials for each form of pain are provided in Supporting Information (Figures S2-1 to S2-3). Sizes of data markers are proportional to the amount of data contributed by each trial. *OR* odds ratio, *CI* confidence interval.

Within the top-six forms of pain, all the trials for each form of pain are given in Supporting Information (Figures S2-1 to S2-3) with the meta-analysis. Depicted in Fig. 1 are only the largest two trials for each form of pain.

A total of 391 bone pain events occurred among 6563 patients in 8 related trials, as seen in Figure S2-1 (upper panel). Within the 8 trials, in each of the largest two trials (Fig. 1A), the treatment group with lapatinib significantly decreased the bone pain events compared to the control group: *P* = 0.0155 for NCT00490139 (with a total of 4137 patients) and *P* = 0.0204 for NCT00073528 (with a total of 1278 patients). For each of the remaining trials, the sample size was too small to demonstrate the significance difference (*P* > 0.05) between the lapatinib group and control group. Overall, according to the meta-analysis, lapatinib was associated with 25% reduction in the odds of bone pain (OR 0.75; 95% CI 0.61–0.93; *P* = 0.0071). Here, OR (Odds Ratio) represents the ratio of odds of the pain event in the lapatinib group to odds of the event in the control group (see Supporting Information for the OR formula) (Figure S2-1, upper panel).

A total of 1449 arthralgia events occurred among 11,268 patients in 15 related trials, as seen in Figure S2-1 (middle panel). Within the 15 trials, in each of the largest two trials (Fig. 1B), the treatment group with lapatinib significantly decreased the arthralgia events compared to the control group: *P* = 0.0001 for NCT00490139 and *P* = 0.0008 for NCT00374322 (with a total of 3147 patients). For each of the remaining trials, the sample size was too small for the arthralgia events to demonstrate significant difference. Overall, lapatinib was associated with 20% reduction in the odds of arthralgia (OR 0.80; 95% CI 0.70–0.93; *P* = 0.0025) (Figure S2-1 middle panel).

A total of 433 musculoskeletal pain events occurred among 7664 patients in 10 related trials, as seen in Figure S2-1 (bottom panel). Within the 10 trials, the largest trial (see Fig. 1C for the largest two trials) was NCT00490139 in which the treatment group with lapatinib significantly decreased the musculoskeletal pain events compared to the control group (*P* = 0.0240). For each of the remaining trials, the difference between the two groups was not statistically significant. Overall, lapatinib was associated with 21% reduction in the odds of musculoskeletal pain (OR 0.79; 95% CI 0.65–0.96; *P* = 0.0189) (Figure S2-1 bottom panel).

A total of 1320 headache events were reported among 12,661 patients in 19 related trials, as shown in Figure S2-2 (upper panel). Within the 19 trials, in each of the largest two trials (Fig. 1D), the treatment group with lapatinib significantly decreased the headache events compared to the control group: *P* = 0.0008 for NCT00490139 and *P* = 0.0085 for NCT00374322. For each of the remaining trials, the sample size was too small for the headache events to demonstrate significant difference. Overall, lapatinib was associated with 19% reduction in the odds of headache (OR 0.81; 95% CI 0.70–0.95; *P* = 0.0078) (Figure S2-2 upper panel).

A total of 597 pain-in-extremity events occurred among 6887 patients in 10 related trials (Figure S2-2, bottom panel). Within the 10 trials, the largest trial NCT00490139 (see Fig. 1E for the largest two trials) revealed that the treatment group with lapatinib significantly decreased the pain-in-extremity events compared to the control group (*P* = 0.0217). For each of the remaining trials, the difference between the two groups was not statistically significant. Overall, lapatinib was associated with 18% reduction in the odds of pain in extremity (OR 0.82; 95% CI 0.70–0.98; *P* = 0.0269) (Figure S2-2, bottom panel).

Notably, within the five forms of pain events discussed above, the largest trial is always NCT00490139 in which the treatment group with lapatinib significantly decreased each form of pain events compared to the control group.

A total of 969 myalgia events occurred among 8121 patients in 14 related trials. In each of these trials, the difference between the two groups in myalgia events was not statistically significant, although the largest trial (NCT00490139, *P* = 0.0596) was close to *P* = 0.05. Overall, lapatinib was associated with 4% reduction in the odds of myalgia (OR 0.96; 95% CI 0.81–1.14; *P* = 0.6647) (Figure S2-3. Interestingly, when only the largest two trials (Fig. 1F) are included in the analysis (ignoring the smaller trials), lapatinib significantly reduced the pain events, with 18% reduction in the odds of myalgia (OR 0.82; 95% CI 0.68–0.98; *P* = 0.0261).

**Figure 2:**
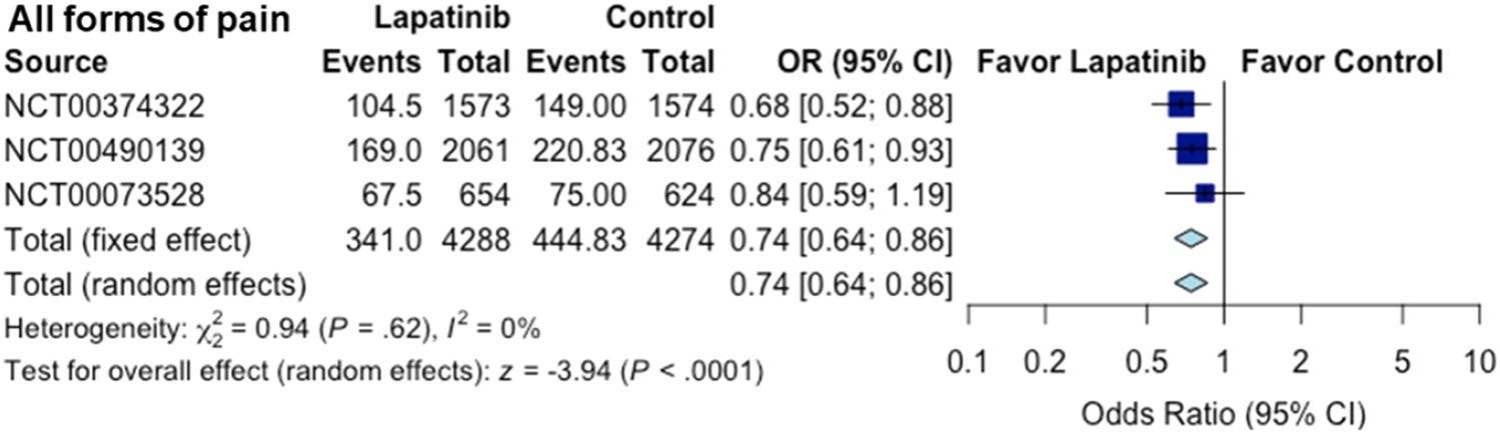
Overall effect of the lapatinib treatment on various forms of pain in all the trials included in Fig. 1. The corresponding figure of the full meta-analysis with all the trials for the six forms of pain is provided in Supporting Information (Figure S2-4). Sizes of data markers are proportional to the amount of data contributed by each trial. *OR* odds ratio, *CI* confidence interval.

When all the above-mentioned data for the six forms of pain were combined, a total of 2370 pain events were recorded among 6435 patients (within the six forms of pain) in the treatment group while there were 2789 pain events (within the six forms of pain) among 6318 patients in the control group. We calculated the average pain event counts in each trial and used this data to analyze the overall analgesic effect. According to the meta-analysis with all the trials (Figure S2-4), lapatinib was associated with a significant 21% reduction in the odds of the major pain events (OR 0.79; CI 0.70–0.89; *P* = 0.0002). When only the largest trials involved in Fig. 1 are included, lapatinib was associated with a significant 26% reduction in the odds of the major pain events (OR 0.74; CI 0.64–0.86; *P* =  < 0.0001) (Fig. 2).

For the above meta-analyses, all *P* values for heterogeneity were greater than 0.1 and all Higgins’ I^2^ values were less than 25%, indicating no significant study bias was observed in the corresponding funnel plots (Figure S3-1 to S3-7).

In the ROBIS analysis, we did not observe any significant source of biases in these reviews. The analysis table can be found in the Supporting Information.

### Lapatinib is an effective pain-relieving agent against various forms of pain

The general analgesic effects on various forms of pain have been clearly manifested in this non-conventional meta-analysis. In all the analyses we observed < 1 OR values, which means that lapatinib can relieve these forms of pain in cancer patients. Besides, in at least five out of six analyses (headache, bone pain, arthralgia, pain in extremity, and musculoskeletal pain), the *P* values were < 0.05, which means that the reduction of OR on these forms of pain was statistically significant.

Both Higgins’ I^2^ and *P* for heterogeneity showed that there was no significant heterogeneity in the trials we used for analysis. Funnel plots (Figure S3-1 to S3-7) also showed there was no significant study bias across these trials, with only one outlier in the arthralgia analysis.

### Other factors have limited impacts on the analgesic effects of lapatinib

As listed in Table 1, the trials involved in the meta-analysis are different on several factors not related to lapatinib, and we would like to know whether these factors impact pain-related outcomes. Hence, we grouped the trials in the overall effect analysis by these other factors to investigate their impacts on the analgesic effects of lapatinib.

As seen in Figure S4-1, the analgesic effects of lapatinib in the masked group (OR 0.79; 95% CI 0.66–0.95) was almost the same as the open label group (OR 0.79; 95% CI 0.67–0.94) with *P* > 0.99, indicating no significant difference.

Similarly, as showed in Figure S4-2, we found that the cancer type of the patient recruited did not have a significant influence on the analgesic effects of lapatinib. Particularly, for gastric cancer patients: OR 0.71; 95% CI 0.27–1.89. For head and neck cancer patients: OR 0.69; 95% CI 0.40–1.18. For breast cancer patients: OR 0.80; 95% CI 0.70–0.91. For cervical cancer patients: OR 0.60; 95% CI 0.20–1.77. For the differences between different subgroups: *P* = 0.91.

As discussed in our previous report^40^, the blood concentration of lapatinib should be well above the necessary concentration to inhibit mPGES-1 under all doses used in these clinical trials, thus the difference should not be distinguishable. As showed in Figure S4-3, the differences between these groups with different doses were very small. Particularly, for 1500 mg/day group: OR 0.80; 95% CI 0.68–0.94. For 1000 mg/day group: OR 0.78; 95% CI 0.64–0.95. For 750 mg/day group: OR 0.83; 95% CI 0.36–1.94. For subgroup differences: *P* = 0.95. These observations agree with our prediction of the analgesic effects of lapatinib.

Although only three trials were purely placebo-controlled, in all the other trials the additional components were present in both the treatment and control groups. Since the existence of lapatinib was the only variable between the experimental and control groups, the effects of lapatinib can still be reasonably evaluated in this case. As showed in Figure S4-4, the analysis indicated that the presence of other drugs had no significant influence on the analgesic effects of lapatinib in all these cases. However, notably, when paclitaxel was also used, lapatinib users had a relatively higher chance to report pain (OR 1.14; 95% CI 0.82–1.60), while in all the other groups the chance of pain was all reduced. When we compared the paclitaxel using group with the pure placebo control group (OR 0.69; 95% CI 0.54–0.89), the difference was statistically significant (Figure S4-5). This phenomenon, however, was not observed when patients took other drugs together with paclitaxel (i.e. when CapeOx was taken together with paclitaxel: OR 0.41; 95% CI 0.16–1.09; when trastuzumab was taken together with paclitaxel: OR 0.83; 95% CI 0.36–1.94). The reason of this interesting observation is not very clear at this point; additional clinical data will be needed to see whether it was just caused by error/uncertainty due to the small sample sizes.

Since these three trials showed significant differences with the other trials, we further tested removing these three trials in the myalgia analysis, and the obtained new results (without these three trials) showed that the pain-relieving effects were statistically significant (Figure S5-1. OR 0.84; 95% CI 0.72–0.99; *P* = 0.0376). We also tested to remove these three trials in the analysis of all other forms of pain, resulting in significant improvement on the statistical significance (*P* value) for all forms of pain (Figure S5-2 to S5-6).

### The analgesic effects of lapatinib are not related to EGFR/HER2 inhibition

Since lapatinib is an FDA-approved drug for cancer treatment, one might wonder whether the analgesic effects of lapatinib were caused by the inhibition of EGFR/HER2 rather than mPGES-1. We investigated the adverse effects data from trial NCT00656136, a phase III trial of another FDA-approved EGFR/HER2 dual inhibitor afatinib^62^. As depicted in Figure S6, although the sample size is small, the data clearly showed that afatinib had no observable analgesic effects. Notably, the data shown in Figure S6 suggest the afatinib treatment actually increased the events of pain in extremity (*P* = 0.0132), whereas the lapatinib decreased the events of pain in extremity according to the above analysis (Figure S2-2, bottom panel). Therefore, we believe that the analgesic effects of lapatinib are not caused by the inhibition of EGFR/HER2.

## Discussion

This CDM study is the first meta-analysis (conducted in a non-conventional way) on the analgesic effects of lapatinib. Conventional methods of meta-analysis have been focused on analysis of available clinical data listed as the primary or secondary outcomes of clinical trials, whereas the required primary or secondary outcomes of clinical trials for the conventional meta-analysis are not available for pain relief with lapatinib. In order to examine the possible analgesic effects of lapatinib, we first collected all available lapatinib-related clinical data about pain beyond the primary and secondary outcomes of clinical trials, and then analyzed the collected clinical data by using a meta-analysis protocol in a non-conventional way. So, our non-conventional meta-analysis was not limited to the primary and secondary outcomes of clinical trials such that more clinical data became available for the meta-analysis. According to our CDM results, lapatinib therapy is associated with a significant reduction in various forms of pain, including musculoskeletal pain, bone pain, headache, arthralgia, and pain in extremity, in cancer patients. Our CDM data have clearly demonstrated the favorable analgesic effects of lapatinib in cancer patients.

Because pain was not considered as a primary or secondary (due to successful anti-cancer effect) outcome during any clinical trial with lapatinib so far, the pain data available is limited to the pain events (i.e. there was pain or not) without any information about the pain severity using any pain scoring system. A pain killer might be able to relieve the pain, but not necessarily be able to completely kill the pain; in such event, the subject would still report the pain event without any pain severity information.

Although the present CDM or non-conventional meta-analysis used clinical data for cancer patients only, it is reasonable to assume that lapatinib will also work similarly on non-cancerous patients with the same forms of pain. Nevertheless, we should try to lower the dose of lapatinib to minimize other known adverse effects.

Further, all the clinical trials analyzed in this study were not originally designed to investigate the analgesic effects. As a result, none of the trials, including that by Kaufman et al.^41^, evaluated the severity of pain (pain intensity with an appropriate pain score). A pain score-based clinical trial could better quantify the efficacy of lapatinib in pain relief, guiding the best possible dosage for analgesic effects to minimize its adverse effects. Besides, all these trials were conducted in cancer patients. Although we have demonstrated that the analgesic effects of lapatinib are not related to its anti-cancer effects and lapatinib is expected to have the similar analgesic effects in non-cancer patients, we still would like to have more direct evidence for the analgesic effects of lapatinib in non-cancer patients with pain. The direct evidence may be provided by conducting a new, sophistically designed clinical trial in a general pain population which may manifest the true analgesic power of lapatinib. Only an appropriately designed clinical trial can verify whether lapatinib can be used to effectively relieve pain. A truly effective non-opioid analgesic would be particularly valuable for chronic pain patients (who suffer pain for at least 3 months) to relief the long-lasting pain.

In addition, it is also necessary to assess any possible side effects of lapatinib in non-cancer patients along with the efficacy study. Concerning commonly experienced occurring adverse effects of lapatinib reported in the oncology trials, in a Phase I dose-escalation study^63^, the highest dose of lapatinib used was 7000 mg per day in twice-daily dosing with no dose-limiting toxicity (DLT) found, demonstrating the safety. On the other hand, lapatinib does have some dose-dependent adverse effects; diarrhea is the most common adverse effect with the high doses. With the commonly used doses (1250 mg and 1500 mg once daily) of lapatinib, diarrhea may occur in some patients within 6 days^14^. If occurs, diarrhea usually lasts 4 to 5 days^64^ before the body has adapted to the drug. Lapatinib-induced diarrhea is usually low-grade (Grade 1, i.e. an increase of less than 4 stools a day)^64^. To balance the possible beneficial and adverse effects of lapatinib for pain relief, one of the primary measures for a clinical trial should be the overall quality of life (QoL) defined by the American Chronic Pain Association (ACPA)^65^. The ACPA-defined QoL is measured by asking each subject to choose the most reasonable one of the 11 descriptions associated with the QoL scale of 0 (the worst QoL: Stay in bed all day; Feel hopeless and helpless about life) to 10 (the best QoL: Go to work/volunteer each day; Normal daily activities each day; Have a social life outside of work; Take an active part in family life). If the analgesic efficacy of lapatinib can be demonstrated, and if there are no intolerable side effects in non-cancer patients as well, lapatinib may be repurposed as a novel type of pain killer to improve the overall QoL of non-cancer patients.

In addition, the general strategy of the CDM or non-conventional meta-analysis used in this study may also be used to examine various therapeutic effects of other drugs beyond the primary and secondary outcomes of the clinical trials.

## Methods

### Data sources and searches

The primary aim of this CDM or non-conventional meta-analysis was to determine the analgesic effects of lapatinib in cancer patients. A comprehensive MEDLINE database search on clinicaltrials.gov was performed to find studies using the search term lapatinib, Tykerb, or GW572016. Data concerning study design, baseline patient characteristics, treatment and results (specifically the Adverse Effects section) were extracted from these reports. All the clinical trials were updated until June 30, 2020.

### Study selection and data extraction

After the comprehensive initial database search, there were 327 related records identified. Because the information about pain was buried in the adverse effect data, trials without adverse effect data posted or without using lapatinib in the treatment group were excluded, leaving 101 studies available for further investigation. Among the remaining trials, non-randomized trials or single-armed trials were also removed, leaving 20 trials for final analysis (Figure S1). Within the 20 trials, the adverse effect data can be obtained from the related publications for 17 trials, and for the remaining three clinical trials the authors only published their partial results as abstracts^45,56,60^. Notably, for all the 20 trials, the adverse effect data have been posted online. Hence, all the pain-related data were collected from clinicaltrials.gov. Since data in the “Serious adverse effects” section across all the trials are all very small (for all forms of pain, no trial has a reported patient count greater than 5, see Table S1), these data were not included in this meta-analysis. Only data from the “Other adverse effects” section were used in this analysis.

### Statistical analysis

The R package meta 4.11-0^66^ was used for all the related analyses and plotting. The Cochrane statistic and Higgins’ I^2^ were calculated for the assessment of heterogeneity across the trials. Odds ratios (OR) and 95% confidence intervals (CI) of various forms of pain were calculated with both fixed and random-effects model (DerSimonian-Laird estimator for τ^2^) from each data point in each study using the Mantel–Haenszel method. The patients allocated to an intervention in a specific trial were only compared with those in the control group of the same trial, avoiding direct comparisons of patients across different trials with other different conditions. *P* value of less than 0.05 was judged as statistically significant. To assess study bias, we generated funnel plots of the logarithm of treatment effects and compared it with their standard errors for each meta-analysis. The ROBIS^67^ was used to assess the risks of bias in this meta-analysis.

It should be noted that possible confounders, such as demographic factors and other pathologies including psychiatric disorders, have not been accounted for in the statistical analysis, because these factors were not considered in the original studies for the pain events. The desirable data for examining these confounders are not available in the original clinical reports.

## Supplementary Information

Supplementary Information.
