# Supplementary material for: Clinical data mining reveals analgesic effects of lapatinib in cancer patients
Source: Sci Rep. 2021 Feb 11;11:3528. doi: 10.1038/s41598-021-82318-w (PMC7878815; doi:10.1038/s41598-021-82318-w)
Supplement: Supplementary file 1 — Supplementary Information. [file 41598_2021_82318_MOESM1_ESM.pdf]

Supporting Information

Clinical data mining reveals analgesic effects of lapatinib in cancer patients

Shuo Zhou,[1,2] Fang Zheng,[1,2,]* and Chang-Guo Zhan[1,2,]*

[1]Molecular Modeling and Biopharmaceutical Center, College of Pharmacy, University of Kentucky, 789 South Limestone Street, Lexington, KY 40536. [2]Department of Pharmaceutical Sciences, College of Pharmacy, University of Kentucky, 789 South Limestone Street, Lexington, KY 40536

Table S1. Data for various forms of pain reported as serious side effects in the clinical trials listed in Table 1. Only data from the six forms of pain discussed in the "Major Pain Events" section are listed here.

Headache

Clinical trial No.	Positive counts in experimental group	Negative counts in experimental group	Positive counts in control group	Negative counts in control group
**NCT00558103**	1	37	0	13
**NCT00374322**	0	1573	1	1573
**NCT00553358**	0	152	1	148
**NCT00490139**	2	2059	1	2075
**NCT00073528**	0	654	2	622
**NCT00390455**	2	139	0	137
**NCT00075270**	1	292	0	286
**NCT00770809**	0	115	1	114

Arthralgia

Clinical trial No.	Positive counts in experimental group	Negative counts in experimental group	Positive counts in control group	Negative counts in control group
**NCT00486954**	1	130	0	129
**NCT00490139**	1	2060	0	2076
**NCT00073528**	2	652	5	619
**NCT00390455**	0	141	1	136
**NCT00075270**	2	291	0	286
**NCT00680901**	0	270	1	266

Bone pain

Clinical trial No.	Positive counts in experimental group	Negative counts in experimental group	Positive counts in control group	Negative counts in control group
**NCT00073528**	1	651	1	623

**NCT00390455**	0	141	1	136
**NCT00374322**	0	1573	1	1573
**NCT00680901**	0	270	1	266
**NCT00770809**	0	115	1	114
**Myalgia**				
**Clinical trial No.**	Positive counts in experimental group	Negative counts in experimental group	Positive counts in control group	Negative counts in control group
**NCT00490139**	2	2059	0	2076
**NCT00075270**	0	293	1	285
**NCT00770809**	3	112	2	113
**NCT00374322**	0	1573	1	1573
**NCT00424255**	1	348	1	335
**Pain in extremity**				
**Clinical trial No.**	Positive counts in experimental group	Negative counts in experimental group	Positive counts in control group	Negative counts in control group
**NCT00073528**	1	653	0	624
**NCT00075270**	3	290	0	286
**Musculoskeletal pain**				
**Clinical trial No.**	Positive counts in experimental group	Negative counts in experimental group	Positive counts in control group	Negative counts in control group
**NCT00073528**	1	653	0	624

Table S2. Analysis of risks of bias about clinical trials used in this meta-analysis.

**Clinical Trial #**	**Random Sequence Generation**	**Blinding of Participants and Personnel**	**Blinding of Outcome Assessment**	**Incomplete Outcome Data**	**Selective Reporting**	**Other Bias**
**NCT00486954**	O	X	X	O	O	O
**NCT00387127**	O	O	O	O	O	O
**NCT00558103**	O	O	O	O	O	X[a]
**NCT00374322**	O	O	O	O	O	O
**NCT00430781**	O	X	X	O	O	O
**NCT00680901**	O	O	O	O	O	O
**NCT00553358**	O	X	X	O	O	O
**NCT00371566**	O	O	X	O	O	X[a]
**NCT00424255**	O	O	O	O	O	O
**NCT00490139**	O	X	X	O	O	O
**NCT00073528**	O	O	O	O	O	O
**NCT00429299**	O	X	X	O	O	O
**NCT00390455**	O	O	O	O	O	O
**NCT00422903**	O	O	O	O	O	O

**NCT00524303**	O	X	X	O	O	O
**NCT00075270**	O	O	O	O	O	O
**NCT00770809**	O	X	X	O	O	O
**NCT00281658**	O	O	O	O	O	O
**NCT00968968**	O	X	X	O	O	O
**NCT01160211**	O	X	X	O	O	O
	a: Patient number in experimental group and control group are not about equal					
**O**	Low Risk of Bias					
**X**	High Risk of Bias					
**?**	Not Enough Information					

Table S3. Patient counts of different kinds of pain tolled this meta-analysis.

Pain Type	Patient Count
**Headache**	12661
**Arthralgia**	11268
**Myalgia**	8420
**Musculoskeletal pain**	7664
**Pain in extremity**	6887
**Bone pain**	6563
**Pharyngolaryngeal pain**	3438
**Chest pain**	2679
**Neck pain**	2191
**Oral pain**	1567
**Skin pain**	1284
**Toothache**	979
**Breast pain**	849
**Ear pain**	750
**Pain in jaw**	579
**Tumor Pain**	579
**Groin Pain**	579
**Lymph node pain**	579
**Pleuritic pain**	579
**Neuralgia**	579
**Procedural Pain**	197
**Pelvic Pain**	150
**Cancer Pain**	51

Table S4. Drug used in experimental group and control group in the 20 trials analyzed

Clinical trial #	Experimental group	Control group
**NCT00486954**	Lapatinib + Paclitaxel	Paclitaxel Alone
**NCT00387127**	Chemoradiotherapy + Lapatinib, Followed by Lapatinib	Chemoradiotherapy + Placebo, Followed by Placebo
**NCT00558103**	Lapatinib + Pazopanib	Pazopanib
**NCT00374322**	Lapatinib	Placebo
**NCT00430781**	Lapatinib + Pazopanib	Pazopanib
**NCT00680901**	CapeOx + Lapatinib	CapeOx + Placebo
**NCT00553358**	Lapatinib + Trastuzumab	Trastuzumab
**NCT00371566**	Lapatinib	Placebo
**NCT00424255**	Lapatinib	Placebo
**NCT00490139**	Lapatinib + Trastuzumab	Trastuzumab
**NCT00073528**	Letrozole + Lapatinib	Letrozole + Placebo
**NCT00429299**	Chemoradiotherapy + Trastuzumab + Lapatinib	Chemotherapy + Trastuzumab
**NCT00390455**	Lapatinib	Placebo
**NCT00422903**	Letrozole + Lapatinib	Letrozole + Placebo
**NCT00524303**	Trastuzumab + Lapatinib	Trastuzumab
**NCT00075270**	Lapatinib + Paclitaxel	Placebo + Paclitaxel
**NCT00770809**	Trastuzumab + Paclitaxel + Lapatinib	Trastuzumab + Paclitaxel
**NCT00281658**	Lapatinib + Paclitaxel	Placebo + Paclitaxel
**NCT00968968**	Lapatinib + Trastuzumab	Trastuzumab
**NCT01160211**	Lapatinib + Trastuzumab + Aromatase Inhibitors	Trastuzumab + Aromatase Inhibitors

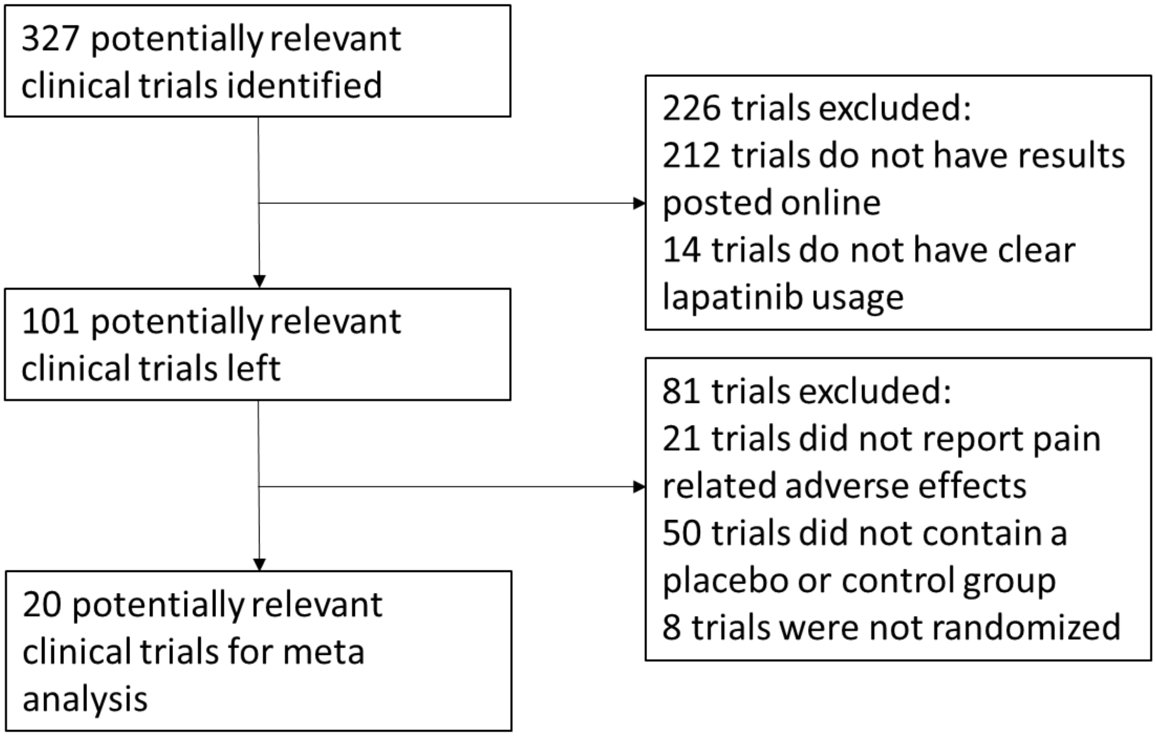

Figure S1. Flow chart for the study selection process of this meta-analysis

Bone Pain

Source	Lapatinib Events	Lapatinib Total	Control Events	Control Total	OR (95% CI)
NCT00490139	88	2061	123	2076	0.71 [0.53; 0.94]
NCT00073528	35	654	54	624	0.60 [0.38; 0.93]
NCT00429299	2	46	2	36	0.77 [0.10; 5.77]
NCT00390455	3	141	2	137	1.47 [0.24; 8.92]
NCT00524303	2	31	1	32	2.14 [0.18; 24.86]
NCT00075270	34	293	32	286	1.04 [0.62; 1.74]
NCT00968968	1	18	0	17	3.00 [0.11; 78.81]
NCT01160211	6	118	6	116	0.98 [0.31; 3.14]
Total (fixed effect)	171	3362	220	3324	0.75 [0.61; 0.93]
Total (random effects)					0.75 [0.61; 0.93]

Heterogeneity: $\chi^2_7 = 4.90$ (P = .67), $I^2 = 0\%$

Test for overall effect (random effects): $z = -2.69$ (P = .0071)

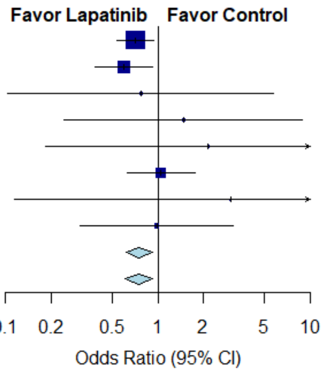

Arthralgia

Source	Lapatinib Events	Lapatinib Total	Control Events	Control Total	OR (95% CI)
NCT00486954	16	131	15	129	1.06 [0.50; 2.24]
NCT00558103	3	38	1	13	1.03 [0.10; 10.85]
NCT00374322	69	1573	113	1574	0.59 [0.44; 0.81]
NCT00430781	5	76	4	74	1.23 [0.32; 4.78]
NCT00553358	9	152	13	149	0.66 [0.27; 1.59]
NCT00490139	306	2061	402	2076	0.73 [0.62; 0.85]
NCT00073528	127	654	145	624	0.80 [0.61; 1.04]
NCT00429299	4	46	1	36	3.33 [0.36; 31.21]
NCT00390455	3	141	6	137	0.47 [0.12; 1.94]
NCT00524303	6	31	5	32	1.30 [0.35; 4.78]
NCT00075270	70	293	58	286	1.23 [0.83; 1.83]
NCT00770809	1	115	1	115	1.00 [0.06; 16.18]
NCT00281658	18	222	14	221	1.30 [0.63; 2.69]
NCT00968968	2	18	3	17	0.58 [0.08; 4.01]
NCT01160211	15	118	14	116	1.06 [0.49; 2.31]
Total (fixed effect)	654	5669	795	5599	0.78 [0.70; 0.87]
Total (random effects)					0.80 [0.70; 0.93]

Heterogeneity: $\chi^2_{14} = 15.61$ (P = .34), $I^2 = 10\%$

Test for overall effect (random effects): $z = -3.02$ (P = .0025)

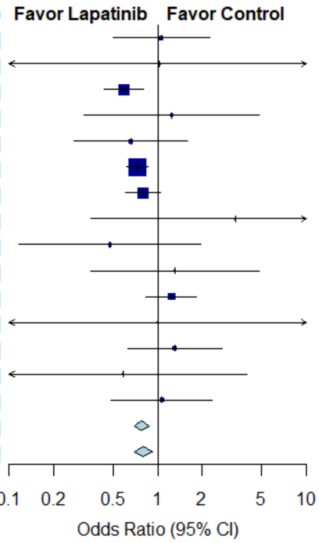

Musculoskeletal Pain

Source	Lapatinib Events	Lapatinib Total	Control Events	Control Total	OR (95% CI)
NCT00558103	0	38	1	13	0.11 [0.00; 2.83]
NCT00553358	4	152	5	149	0.78 [0.20; 2.96]
NCT00424255	15	349	23	336	0.61 [0.31; 1.19]
NCT00490139	79	2061	110	2076	0.71 [0.53; 0.96]
NCT00073528	52	654	56	624	0.88 [0.59; 1.30]
NCT00422903	6	43	9	49	0.72 [0.23; 2.22]
NCT00524303	2	31	0	32	5.51 [0.25; 119.50]
NCT00075270	23	293	27	286	0.82 [0.46; 1.46]
NCT00281658	10	222	6	221	1.69 [0.60; 4.73]
NCT00968968	4	18	1	17	4.57 [0.46; 45.86]
Total (fixed effect)	195	3861	238	3803	0.80 [0.65; 0.97]
Total (random effects)					0.79 [0.65; 0.96]

Heterogeneity: $\chi^2_9 = 8.62$ (P = .47), $I^2 = 0\%$

Test for overall effect (random effects): $z = -2.35$ (P = .0189)

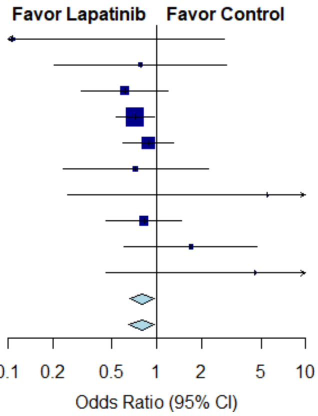

Figure S2-1. Overall effect of lapatinib treatment on bone pain, arthralgia, and musculoskeletal pain. Sizes of data markers are proportional to the amount of data contributed by each trial. OR, odds ratio; CI, confidence interval.

Headache

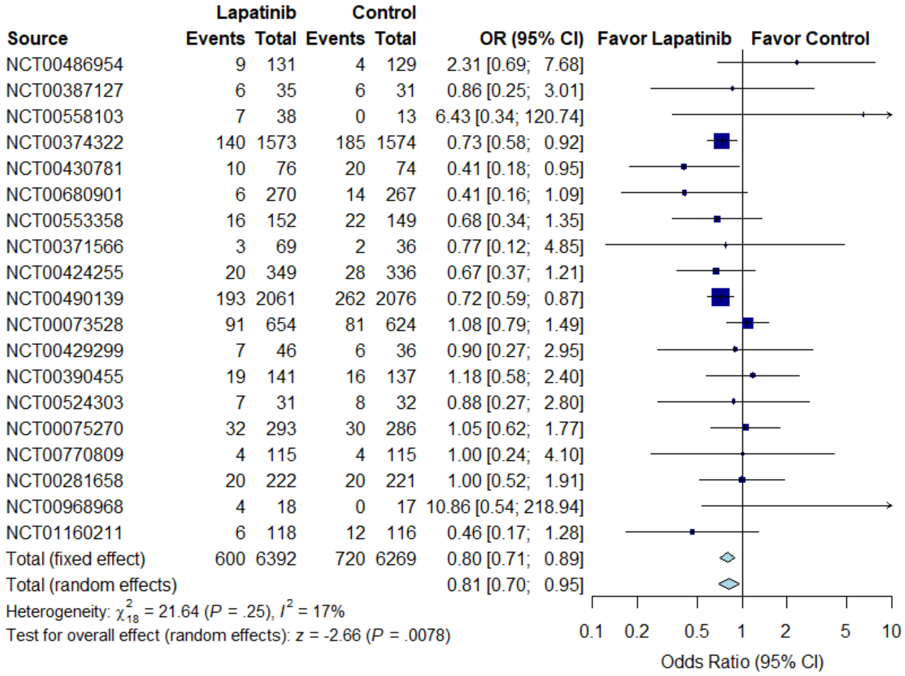

Pain in Extremity

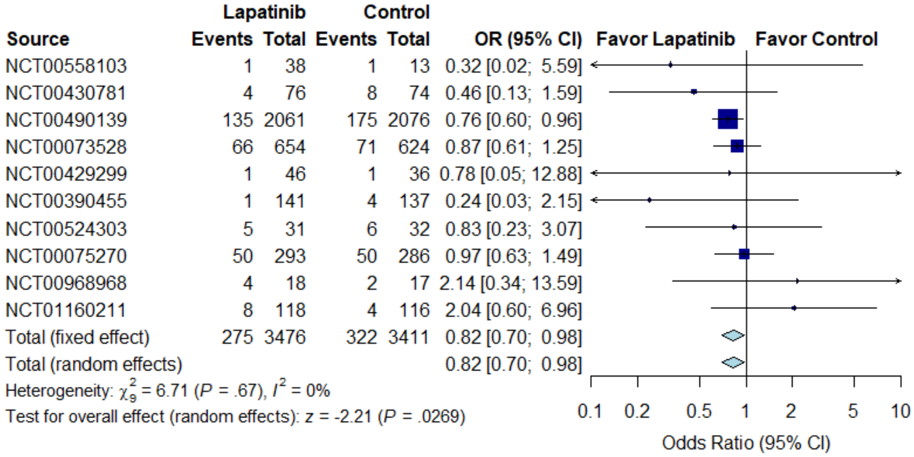

Figure S2-2. Overall effect of lapatinib treatment on headache and pain in extremity. Sizes of data markers are proportional to the amount of data contributed by each trial. OR, odds ratio; CI, confidence interval.

Myalgia

Source	Lapatinib Events	Lapatinib Total	Control Events	Control Total	OR (95% CI)
NCT00486954	21	131	22	129	0.93 [0.48; 1.79]
NCT00558103	6	38	1	13	2.25 [0.24; 20.69]
NCT00430781	5	76	5	74	0.97 [0.27; 3.51]
NCT00553358	24	152	25	149	0.93 [0.50; 1.72]
NCT00490139	213	2061	253	2076	0.83 [0.68; 1.01]
NCT00073528	34	654	43	624	0.74 [0.47; 1.18]
NCT00429299	2	46	1	36	1.59 [0.14; 18.27]
NCT00390455	0	141	1	137	0.32 [0.01; 7.96]
NCT00524303	9	31	4	32	2.86 [0.78; 10.54]
NCT00075270	94	293	74	286	1.35 [0.94; 1.94]
NCT00770809	28	115	34	115	0.77 [0.43; 1.38]
NCT00281658	30	222	23	221	1.35 [0.75; 2.40]
NCT00968968	3	18	0	17	7.90 [0.38; 165.39]
NCT01160211	6	118	8	116	0.72 [0.24; 2.15]
Total (fixed effect)	475	4096	494	4025	0.94 [0.82; 1.08]
Total (random effects)					0.96 [0.81; 1.14]

Heterogeneity: $\chi^2_{13} = 14.56$ ($P = .34$), $I^2 = 11\%$

Test for overall effect (random effects): $z = -0.43$ ($P = .6647$)

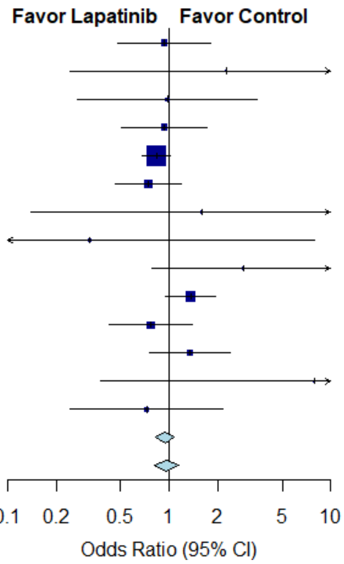

Figure S2-3. Overall effect of lapatinib treatment on myalgia. Sizes of data markers are proportional to the amount of data contributed by each trial. OR, odds ratio; CI, confidence interval.

Source	Lapatinib Events	Lapatinib Total	Control Events	Control Total	OR (95% CI)
NCT00486954	15.33	131	13.67	129	1.12 [0.52; 2.42]
NCT00387127	6.00	35	6.00	31	0.86 [0.25; 3.01]
NCT00558103	3.40	38	0.80	13	1.50 [0.12; 18.65]
NCT00374322	104.50	1573	149.00	1574	0.68 [0.52; 0.88]
NCT00430781	6.00	76	9.25	74	0.60 [0.20; 1.77]
NCT00680901	6.00	270	14.00	267	0.41 [0.16; 1.09]
NCT00553358	13.25	152	16.25	149	0.78 [0.36; 1.67]
NCT00371566	3.00	69	2.00	36	0.77 [0.12; 4.85]
NCT00424255	17.50	349	25.50	336	0.64 [0.34; 1.20]
NCT00490139	169.00	2061	220.83	2076	0.75 [0.61; 0.93]
NCT00073528	67.50	654	75.00	624	0.84 [0.59; 1.19]
NCT00429299	3.20	46	2.20	36	1.15 [0.19; 6.78]
NCT00390455	5.20	141	5.80	137	0.87 [0.26; 2.90]
NCT00422903	6.00	43	9.00	49	0.72 [0.23; 2.22]
NCT00524303	5.17	31	4.00	32	1.40 [0.34; 5.74]
NCT00075270	50.50	293	45.17	286	1.11 [0.72; 1.72]
NCT00770809	11.00	115	13.00	115	0.83 [0.36; 1.94]
NCT00281658	19.50	222	15.75	221	1.25 [0.63; 2.51]
NCT00968968	3.00	18	1.00	17	3.20 [0.30; 34.24]
NCT01160211	8.20	118	8.80	116	0.91 [0.34; 2.44]
Total (fixed effect)	523.25	6435	637.02	6318	0.79 [0.70; 0.89]
Total (random effects)					0.79 [0.70; 0.89]

Heterogeneity: $\chi^2_{19} = 11.37$ ($P = .91$), $I^2 = 0\%$

Test for overall effect (random effects): $z = -3.77$ ($P = .0002$)

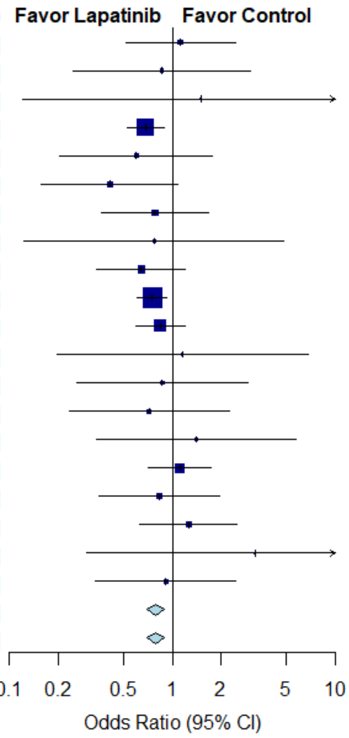

Figure S2-4. Overall effect of the lapatinib treatment on various forms of pain. Sizes of data markers are proportional to the amount of data contributed by each trial. OR, odds ratio; CI, confidence interval.

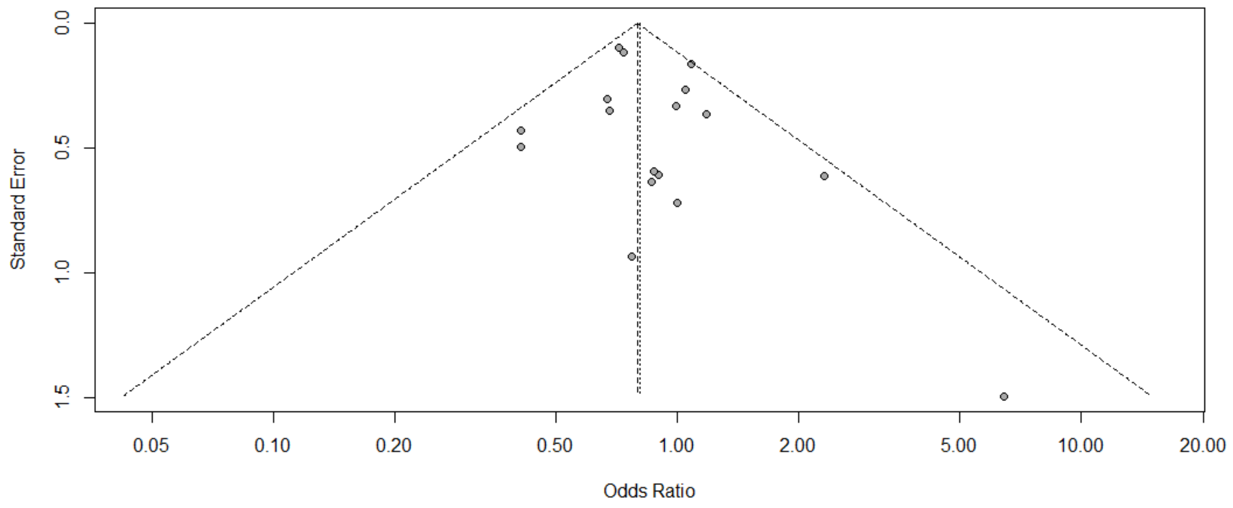

Figure S3-1. Funnel plot for headache

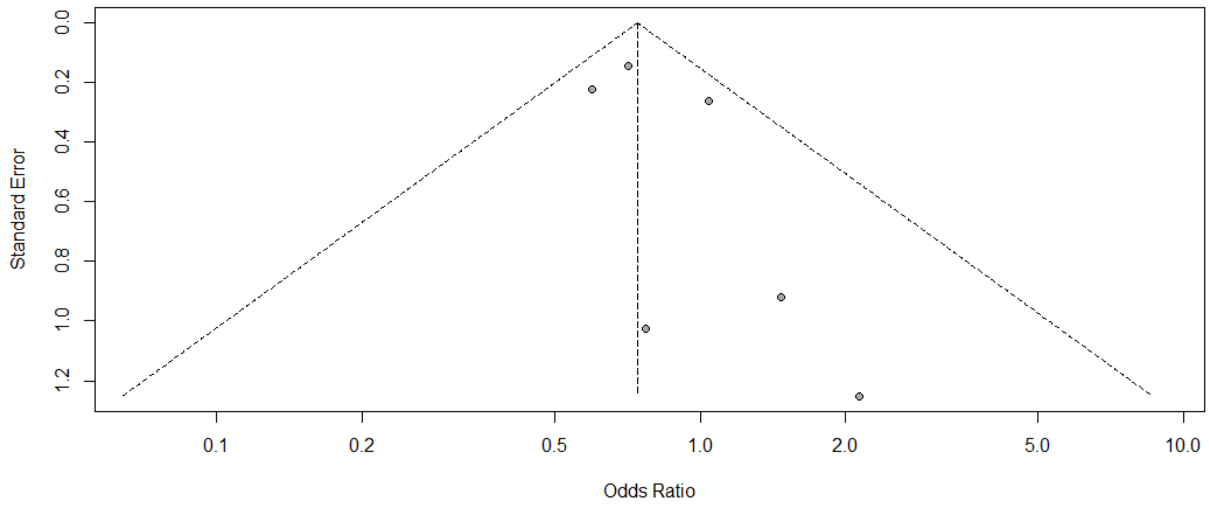

Figure S3-2. Funnel plot for bone pain

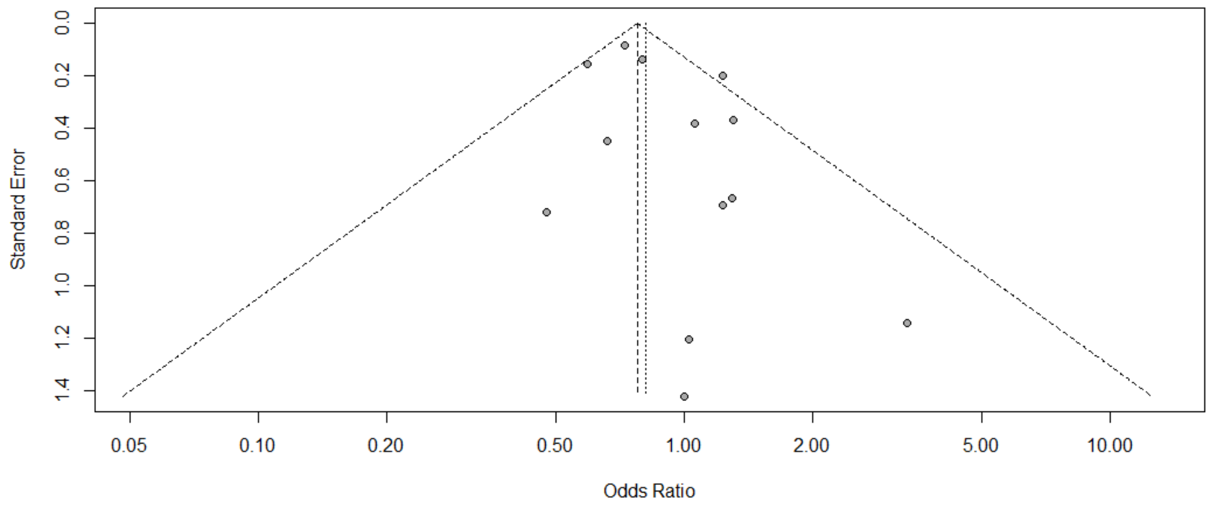

Figure S3-3. Funnel plot for arthralgia

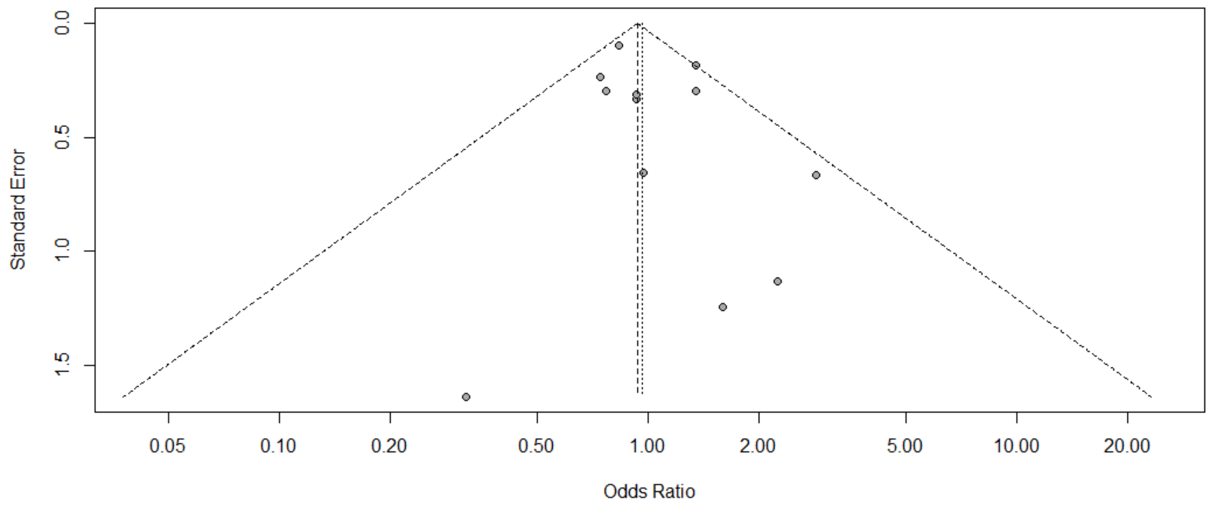

Figure S3-4. Funnel plot for myalgia

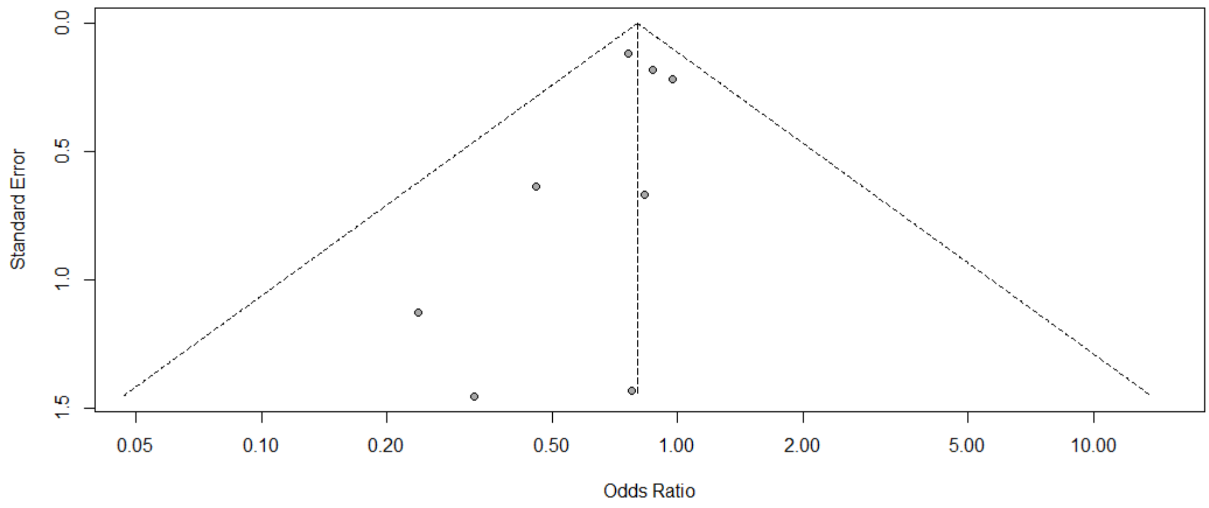

Figure S3-5. Funnel plot for pain in extremity

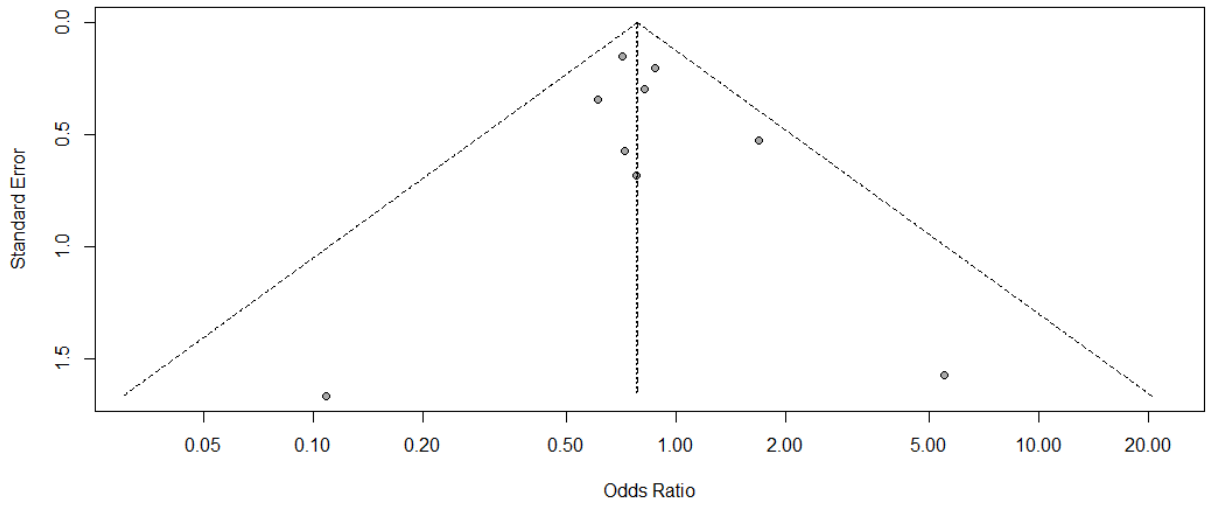

Figure S3-6. Funnel plot for musculoskeletal pain

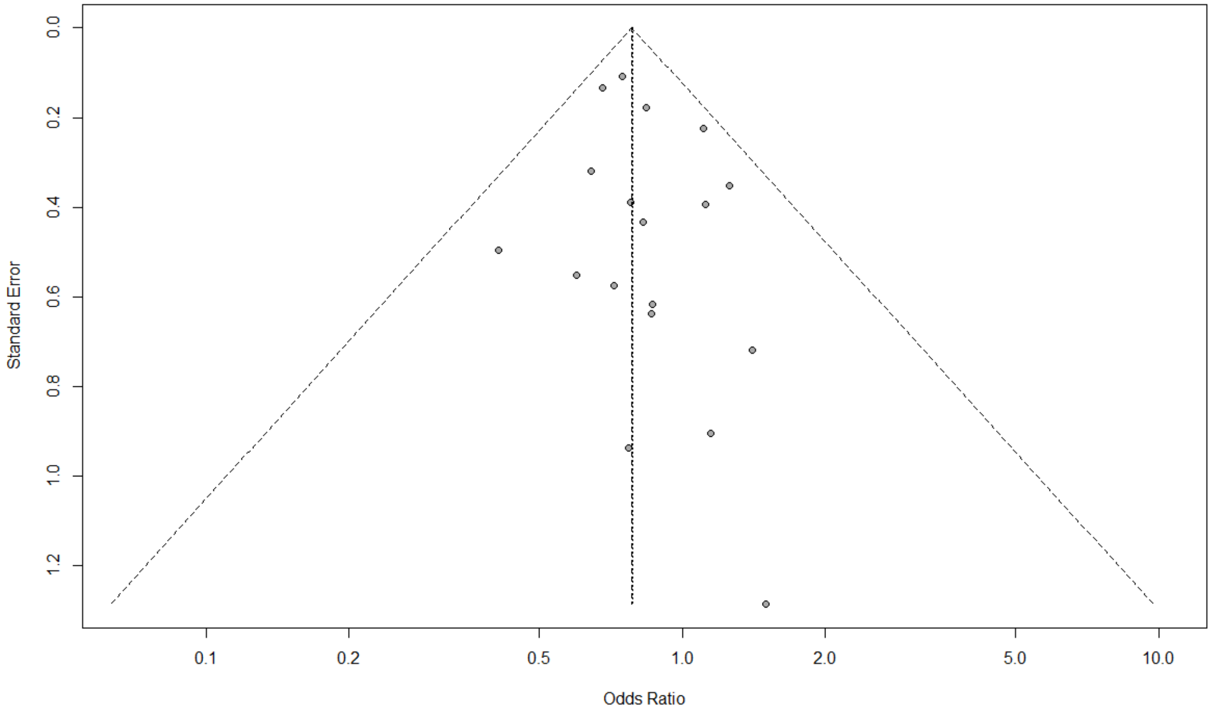

Figure S3-7. Funnel plot for the overall effect analysis

Source	Lapatinib Events	Lapatinib Total	Control Events	Control Total	OR (95% CI)
**Masked = FALSE**					
NCT00486954	15.33	131	13.67	129	1.12 [0.52; 2.42]
NCT00430781	6.00	76	9.25	74	0.60 [0.20; 1.77]
NCT00553358	13.25	152	16.25	149	0.78 [0.36; 1.67]
NCT00490139	169.00	2061	220.83	2076	0.75 [0.61; 0.93]
NCT00429299	3.20	46	2.20	36	1.15 [0.19; 6.78]
NCT00524303	5.17	31	4.00	32	1.40 [0.34; 5.74]
NCT00770809	11.00	115	13.00	115	0.83 [0.36; 1.94]
NCT00968968	3.00	18	1.00	17	3.20 [0.30; 34.24]
NCT01160211	8.20	118	8.80	116	0.91 [0.34; 2.44]
Total	234.15	2748	289.00	2744	0.79 [0.66; 0.95]
Heterogeneity: $\chi^2_8 = 3.49$ (P = .90), $I^2 = 0\%$					
**Masked = TRUE**					
NCT00387127	6.00	35	6.00	31	0.86 [0.25; 3.01]
NCT00558103	3.40	38	0.80	13	1.50 [0.12; 18.65]
NCT00374322	104.50	1573	149.00	1574	0.68 [0.52; 0.88]
NCT00680901	6.00	270	14.00	267	0.41 [0.16; 1.09]
NCT00371566	3.00	69	2.00	36	0.77 [0.12; 4.85]
NCT00424255	17.50	349	25.50	336	0.64 [0.34; 1.20]
NCT00073528	67.50	654	75.00	624	0.84 [0.59; 1.19]
NCT00390455	5.20	141	5.80	137	0.87 [0.26; 2.90]
NCT00422903	6.00	43	9.00	49	0.72 [0.23; 2.22]
NCT00075270	50.50	293	45.17	286	1.11 [0.72; 1.72]
NCT00281658	19.50	222	15.75	221	1.25 [0.63; 2.51]
Total	289.10	3687	348.02	3574	0.79 [0.67; 0.93]
Heterogeneity: $\chi^2_{10} = 7.89$ (P = .64), $I^2 = 0\%$					

Heterogeneity: $\chi^2_{19} = 11.37$ (P = .91), $I^2 = 0\%$

Test for subgroup differences: $\chi^2_1 = 0.00$ (P > .99)

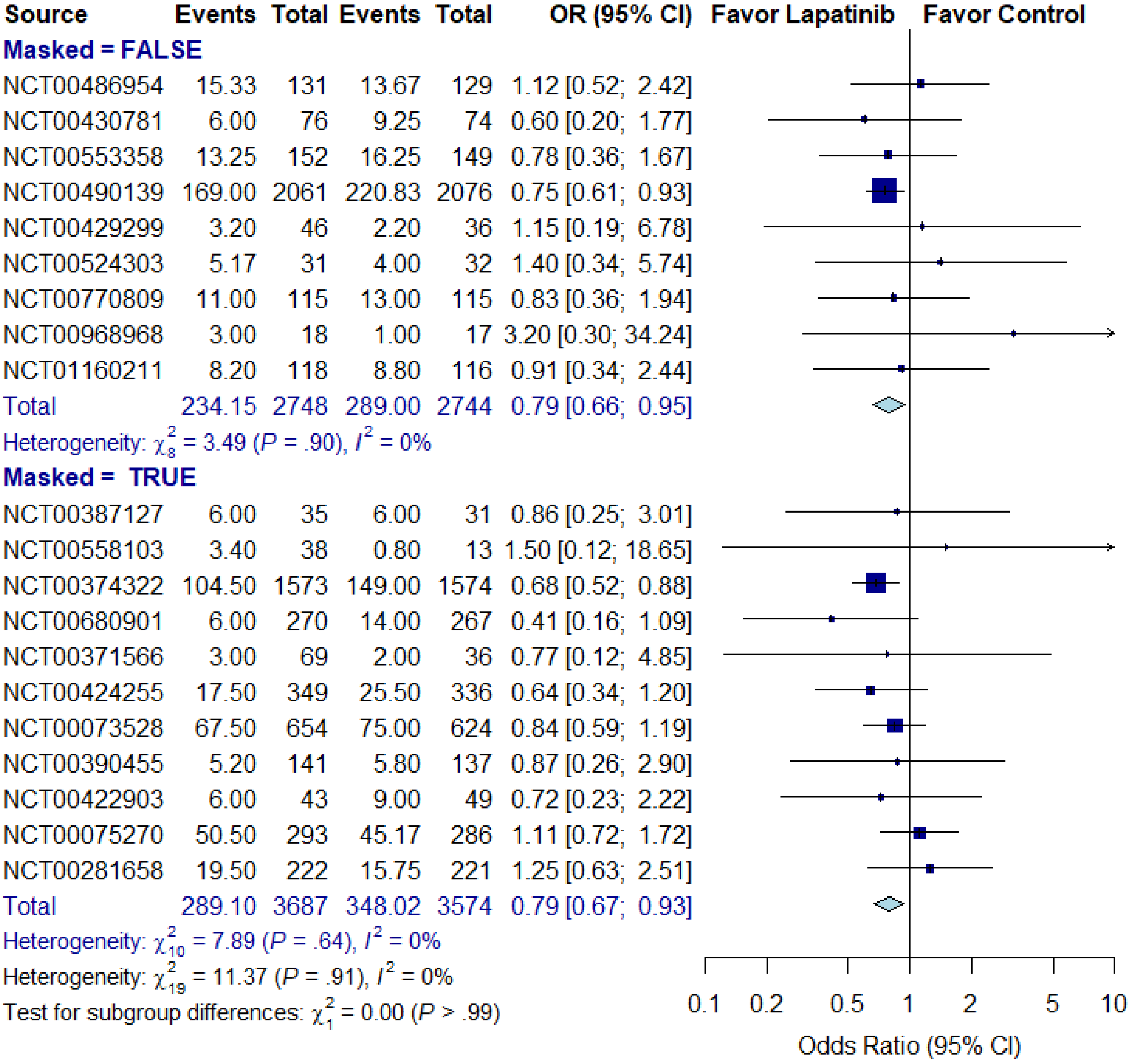

Figure S4-1. Overall pain-relieving effect of lapatinib, grouped by the factor concerning whether the trials were masked or not.

Source	Lapatinib Events	Lapatinib Total	Control Events	Control Total	OR (95% CI)
**Cancer Type = Gastric Cancer**					
NCT00486954	15.33	131	13.67	129	1.12 [0.52; 2.42]
NCT00680901	6.00	270	14.00	267	0.41 [0.16; 1.09]
Total	21.33	401	27.67	396	0.71 [0.27; 1.89]
Heterogeneity: χ^2_1 = 2.5 (*P* = .11), I^2 = 60%					
**Cancer Type = Head and Neck Cancer**					
NCT00387127	6.00	35	6.00	31	0.86 [0.25; 3.01]
NCT00371566	3.00	69	2.00	36	0.77 [0.12; 4.85]
NCT00424255	17.50	349	25.50	336	0.64 [0.34; 1.20]
Total	26.50	453	33.50	403	0.69 [0.40; 1.18]
Heterogeneity: χ^2_2 = 0.19 (*P* = .91), I^2 = 0%					
**Cancer Type = Breast Cancer**					
NCT00558103	3.40	38	0.80	13	1.50 [0.12; 18.65]
NCT00374322	104.50	1573	149.00	1574	0.68 [0.52; 0.88]
NCT00553358	13.25	152	16.25	149	0.78 [0.36; 1.67]
NCT00490139	169.00	2061	220.83	2076	0.75 [0.61; 0.93]
NCT00073528	67.50	654	75.00	624	0.84 [0.59; 1.19]
NCT00429299	3.20	46	2.20	36	1.15 [0.19; 6.78]
NCT00390455	5.20	141	5.80	137	0.87 [0.26; 2.90]
NCT00422903	6.00	43	9.00	49	0.72 [0.23; 2.22]
NCT00524303	5.17	31	4.00	32	1.40 [0.34; 5.74]
NCT00075270	50.50	293	45.17	286	1.11 [0.72; 1.72]
NCT00770809	11.00	115	13.00	115	0.83 [0.36; 1.94]
NCT00281658	19.50	222	15.75	221	1.25 [0.63; 2.51]
NCT00968968	3.00	18	1.00	17	3.20 [0.30; 34.24]
NCT01160211	8.20	118	8.80	116	0.91 [0.34; 2.44]
Total	469.42	5505	566.60	5445	0.80 [0.70; 0.91]
Heterogeneity: χ^2_{13} = 8.13 (*P* = .83), I^2 = 0%					
**Cancer Type = Cervical Cancer**					
NCT00430781	6.00	76	9.25	74	0.60 [0.20; 1.77]
Total	6.00	76	9.25	74	0.60 [0.20; 1.77]
Heterogeneity: not applicable					
Heterogeneity: χ^2_{19} = 11.37 (*P* = .91), I^2 = 0%					
Test for subgroup differences: χ^2_3 = 0.59 (*P* = .90)					

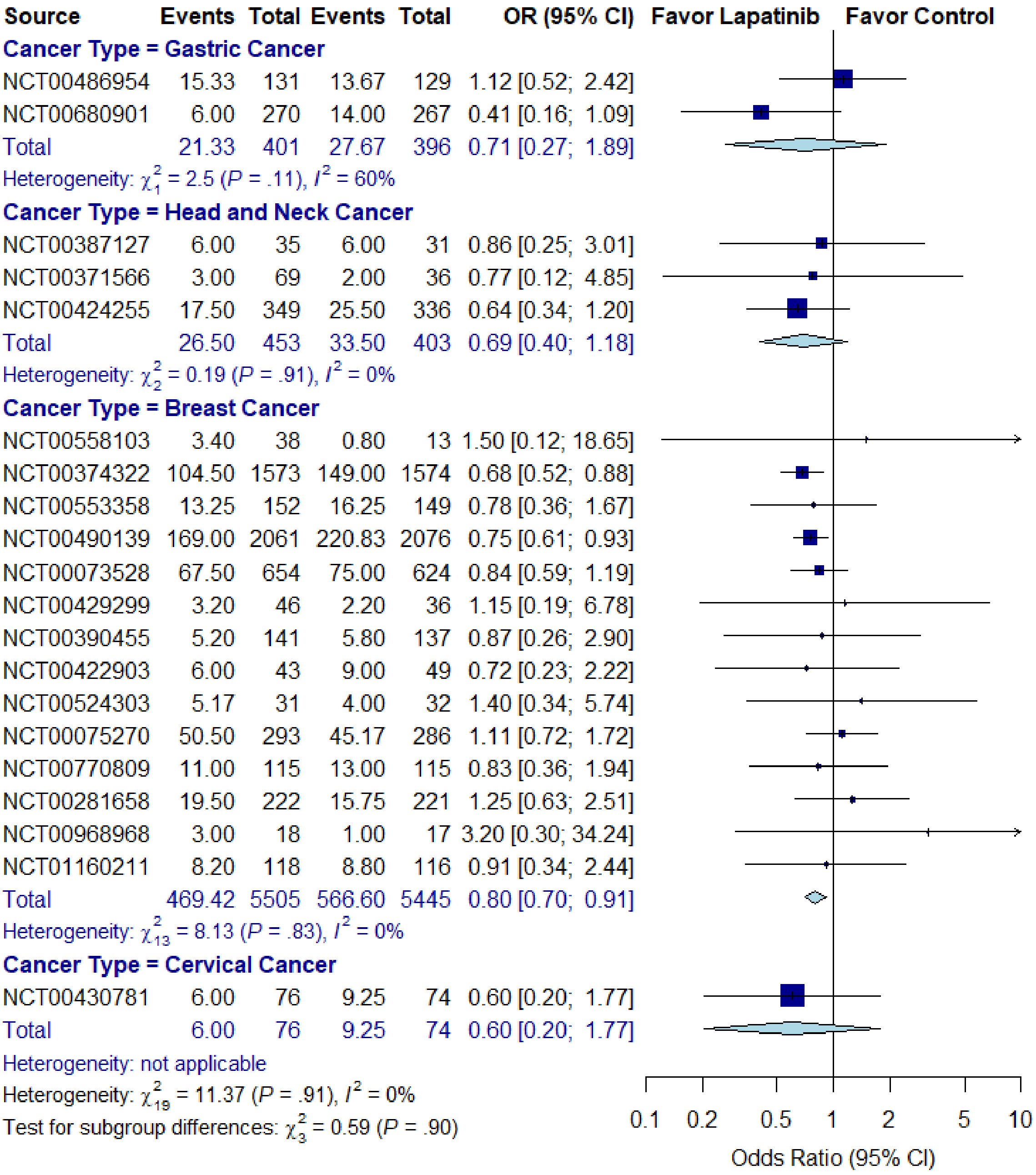

Figure S4-2. Overall pain-relieving effect of lapatinib, grouped by the cancer type of the recruited patients.

Source	Lapatinib Events	Lapatinib Total	Control Events	Control Total	OR (95% CI)
**Dosage = 1500mg**					
NCT00486954	15.33	131	13.67	129	1.12 [0.52; 2.42]
NCT00387127	6.00	35	6.00	31	0.86 [0.25; 3.01]
NCT00558103	3.40	38	0.80	13	1.50 [0.12; 18.65]
NCT00374322	104.50	1573	149.00	1574	0.68 [0.52; 0.88]
NCT00430781	6.00	76	9.25	74	0.60 [0.20; 1.77]
NCT00680901	6.00	270	14.00	267	0.41 [0.16; 1.09]
NCT00371566	3.00	69	2.00	36	0.77 [0.12; 4.85]
NCT00424255	17.50	349	25.50	336	0.64 [0.34; 1.20]
NCT00073528	67.50	654	75.00	624	0.84 [0.59; 1.19]
NCT00390455	5.20	141	5.80	137	0.87 [0.26; 2.90]
NCT00422903	6.00	43	9.00	49	0.72 [0.23; 2.22]
NCT00075270	50.50	293	45.17	286	1.11 [0.72; 1.72]
NCT00281658	19.50	222	15.75	221	1.25 [0.63; 2.51]
Total	310.43	3894	370.94	3777	0.80 [0.68; 0.94]
Heterogeneity: $\chi^2_{12} = 8.9$ ($P = .71$), $I^2 = 0\%$					
**Dosage = 1000mg**					
NCT00553358	13.25	152	16.25	149	0.78 [0.36; 1.67]
NCT00490139	169.00	2061	220.83	2076	0.75 [0.61; 0.93]
NCT00429299	3.20	46	2.20	36	1.15 [0.19; 6.78]
NCT00524303	5.17	31	4.00	32	1.40 [0.34; 5.74]
NCT00968968	3.00	18	1.00	17	3.20 [0.30; 34.24]
NCT01160211	8.20	118	8.80	116	0.91 [0.34; 2.44]
Total	201.82	2426	253.08	2426	0.78 [0.64; 0.95]
Heterogeneity: $\chi^2_{5} = 2.43$ ($P = .79$), $I^2 = 0\%$					
**Dosage = 750mg**					
NCT00770809	11.00	115	13.00	115	0.83 [0.36; 1.94]
Total	11.00	115	13.00	115	0.83 [0.36; 1.94]
Heterogeneity: not applicable					

Heterogeneity: $\chi^2_{19} = 11.37$ ($P = .91$), $I^2 = 0\%$

Test for subgroup differences: $\chi^2_{2} = 0.05$ ($P = .98$)

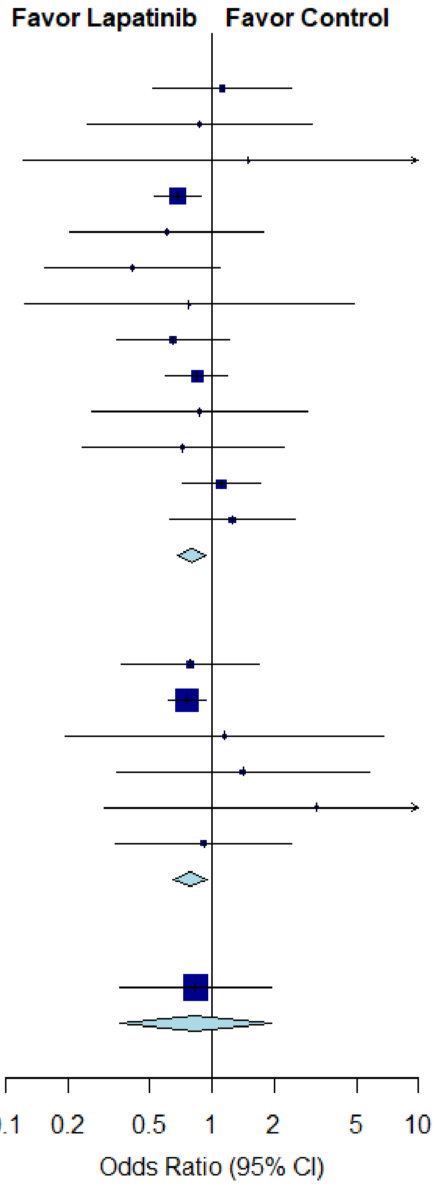

Figure S4-3. Overall pain-relieving effect of lapatinib, grouped by the dosage of lapatinib used in these trials.

Source	Lapatinib Events	Lapatinib Total	Control Events	Control Total	OR (95% CI)
**Additional Component = Paclitaxel**					
NCT00486954	15.33	131	13.67	129	1.12 [0.52; 2.42]
NCT00075270	50.50	293	45.17	286	1.11 [0.72; 1.72]
NCT00281658	19.50	222	15.75	221	1.25 [0.63; 2.51]
Total	85.33	646	74.59	636	1.14 [0.82; 1.60]
Heterogeneity: $\chi^2_2 = 0.09$ ($P = .96$), $I^2 = 0\%$					
**Additional Component = Radiotherapy**					
NCT00387127	6.00	35	6.00	31	0.86 [0.25; 3.01]
NCT00424255	17.50	349	25.50	336	0.64 [0.34; 1.20]
Total	23.50	384	31.50	367	0.68 [0.39; 1.20]
Heterogeneity: $\chi^2_1 = 0.17$ ($P = .68$), $I^2 = 0\%$					
**Additional Component = Pazopanib**					
NCT00558103	3.40	38	0.80	13	1.50 [0.12; 18.65]
NCT00430781	6.00	76	9.25	74	0.60 [0.20; 1.77]
Total	9.40	114	10.05	87	0.69 [0.26; 1.87]
Heterogeneity: $\chi^2_1 = 0.43$ ($P = .51$), $I^2 = 0\%$					
**Additional Component = Placebo**					
NCT00374322	104.50	1573	149.00	1574	0.68 [0.52; 0.88]
NCT00371566	3.00	69	2.00	36	0.77 [0.12; 4.85]
NCT00390455	5.20	141	5.80	137	0.87 [0.26; 2.90]
Total	112.70	1783	156.80	1747	0.69 [0.54; 0.89]
Heterogeneity: $\chi^2_2 = 0.16$ ($P = .92$), $I^2 = 0\%$					
**Additional Component = Paclitaxel+CapeOx**					
NCT00680901	6.00	270	14.00	267	0.41 [0.16; 1.09]
Total	6.00	270	14.00	267	0.41 [0.16; 1.09]
Heterogeneity: not applicable					
**Additional Component = Trastuzumab**					
NCT00553358	13.25	152	16.25	149	0.78 [0.36; 1.67]
NCT00490139	169.00	2061	220.83	2076	0.75 [0.61; 0.93]
NCT00429299	3.20	46	2.20	36	1.15 [0.19; 6.78]
NCT00524303	5.17	31	4.00	32	1.40 [0.34; 5.74]
NCT00968968	3.00	18	1.00	17	3.20 [0.30; 34.24]
Total	193.62	2308	244.28	2310	0.77 [0.63; 0.94]
Heterogeneity: $\chi^2_4 = 2.33$ ($P = .67$), $I^2 = 0\%$					
**Additional Component = Letrozole**					
NCT00073528	67.50	654	75.00	624	0.84 [0.59; 1.19]
NCT00422903	6.00	43	9.00	49	0.72 [0.23; 2.22]
Total	73.50	697	84.00	673	0.83 [0.60; 1.16]
Heterogeneity: $\chi^2_1 = 0.07$ ($P = .80$), $I^2 = 0\%$					
**Additional Component = Paclitaxel+Trastuzumab**					
NCT00770809	11.00	115	13.00	115	0.83 [0.36; 1.94]
Total	11.00	115	13.00	115	0.83 [0.36; 1.94]
Heterogeneity: not applicable					
**Additional Component = Trastuzumab+AI**					
NCT01160211	8.20	118	8.80	116	0.91 [0.34; 2.44]
Total	8.20	118	8.80	116	0.91 [0.34; 2.44]
Heterogeneity: not applicable					
Heterogeneity: $\chi^2_{19} = 11.37$ ($P = .91$), $I^2 = 0\%$					
Test for subgroup differences: $\chi^2_8 = 8.13$ ($P = .42$)					

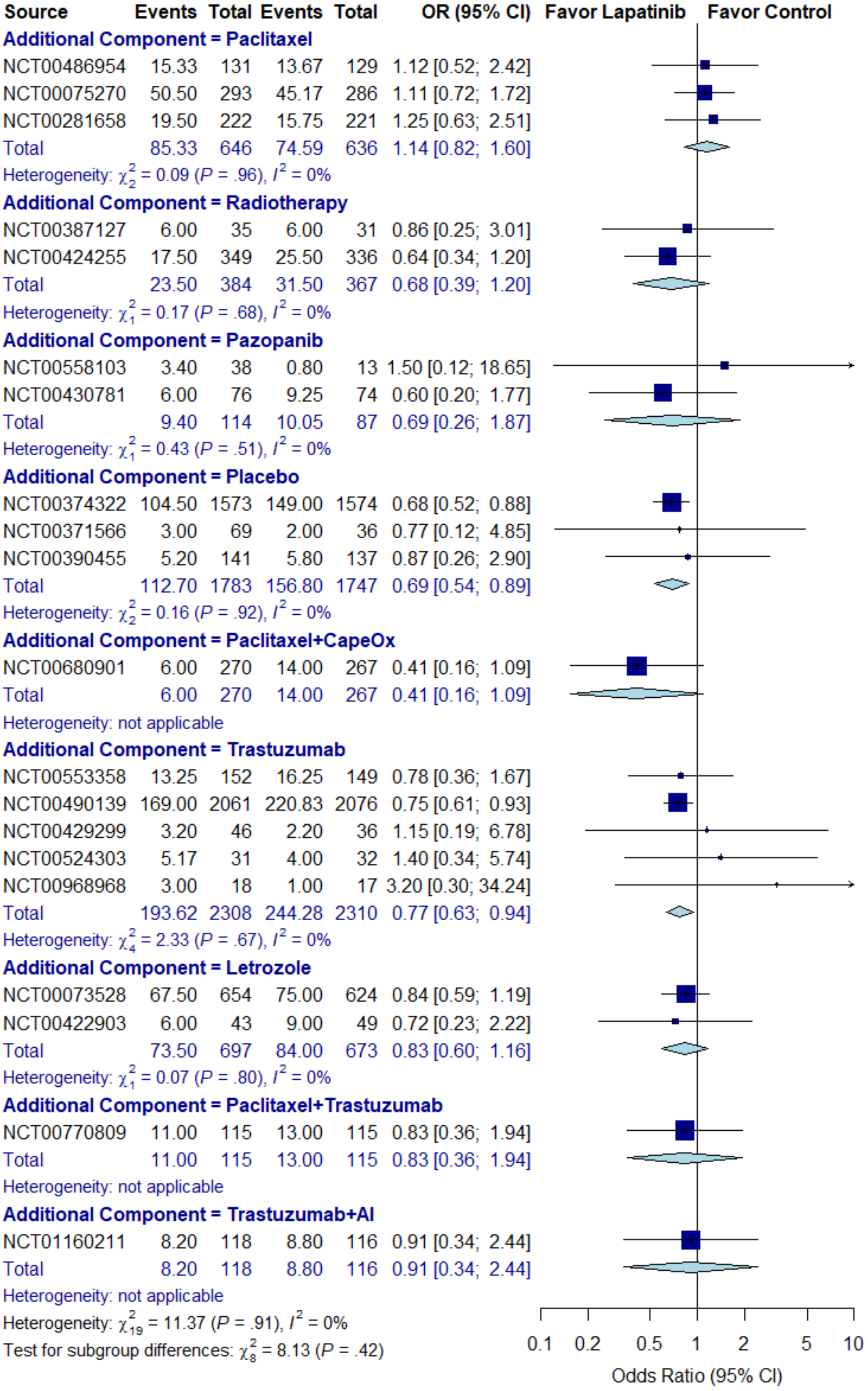

Figure S4-4. Overall pain-relieving effect of lapatinib, grouped by the additional components involved in these trials.

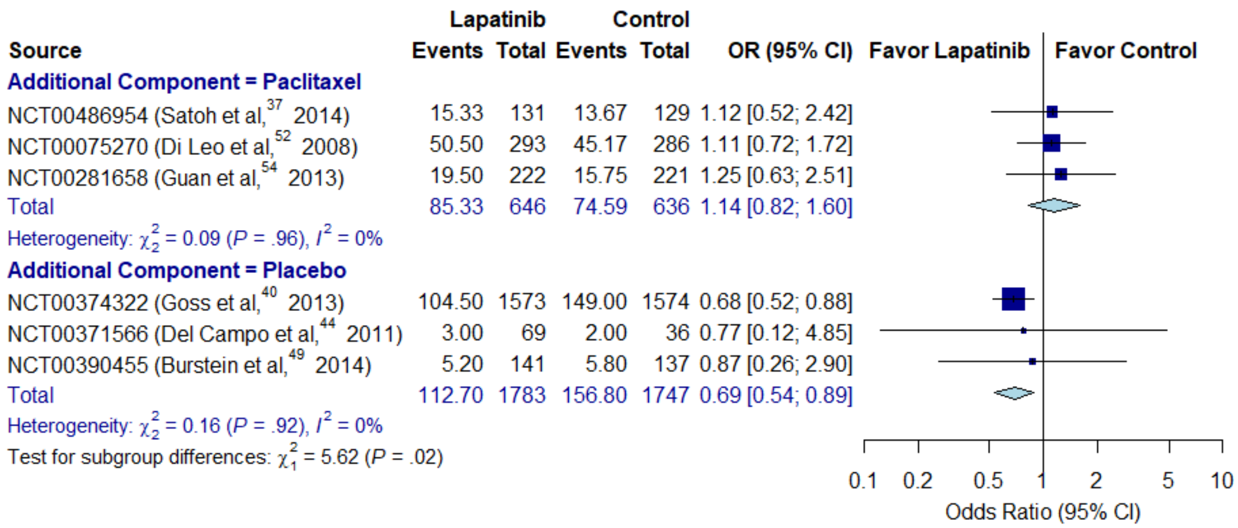

Figure S4-5. Overall pain-relieving effect of lapatinib, comparing only the group with paclitaxel used and the pure placebo control group.

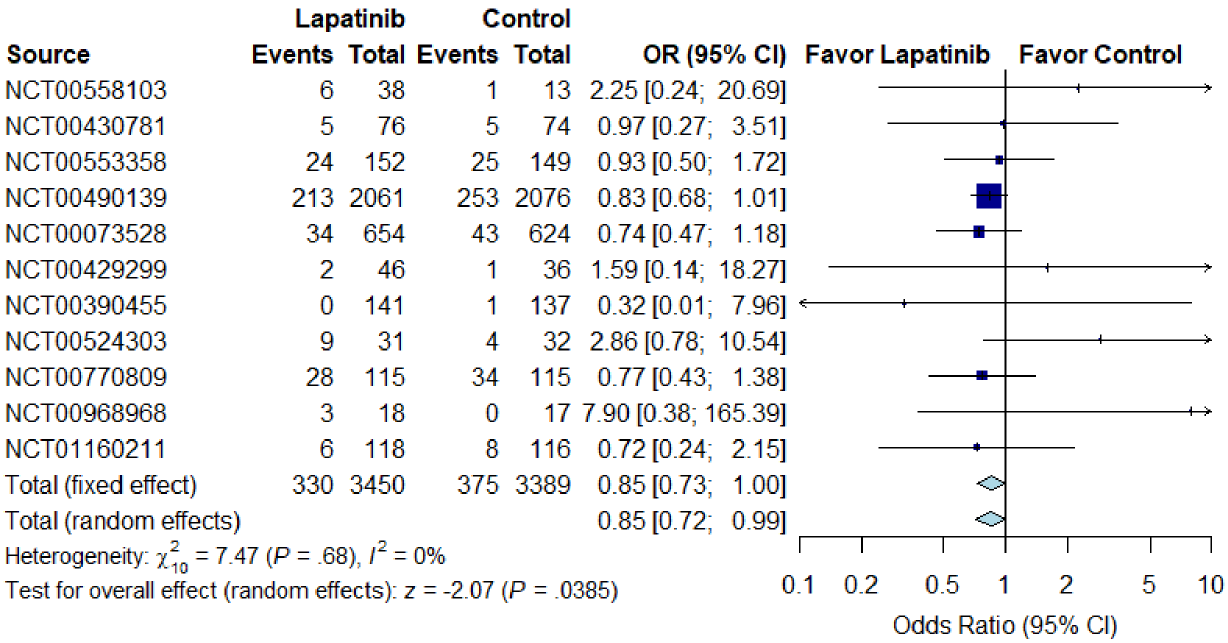

Figure S5-1. Effect of lapatinib on myalgia, when NCT00075270, NCT00486954, and NCT00281658 were excluded.

Source	Lapatinib Events	Lapatinib Total	Control Events	Control Total	OR (95% CI)
NCT00387127	6	35	6	31	0.86 [0.25; 3.01]
NCT00558103	7	38	0	13	6.43 [0.34; 120.74]
NCT00374322	140	1573	185	1574	0.73 [0.58; 0.92]
NCT00430781	10	76	20	74	0.41 [0.18; 0.95]
NCT00680901	6	270	14	267	0.41 [0.16; 1.09]
NCT00553358	16	152	22	149	0.68 [0.34; 1.35]
NCT00371566	3	69	2	36	0.77 [0.12; 4.85]
NCT00424255	20	349	28	336	0.67 [0.37; 1.21]
NCT00490139	193	2061	262	2076	0.72 [0.59; 0.87]
NCT00073528	91	654	81	624	1.08 [0.79; 1.49]
NCT00429299	7	46	6	36	0.90 [0.27; 2.95]
NCT00390455	19	141	16	137	1.18 [0.58; 2.40]
NCT00524303	7	31	8	32	0.88 [0.27; 2.80]
NCT00770809	4	115	4	115	1.00 [0.24; 4.10]
NCT00968968	4	18	0	17	10.86 [0.54; 218.94]
NCT01160211	6	118	12	116	0.46 [0.17; 1.28]
Total (fixed effect)	539	5746	666	5633	0.77 [0.68; 0.87]
Total (random effects)					0.77 [0.66; 0.89]

Heterogeneity: χ^2_{15} = 16.76 (P = .33), I^2 = 10%

Test for overall effect (random effects): z = -3.42 (P = .0006)

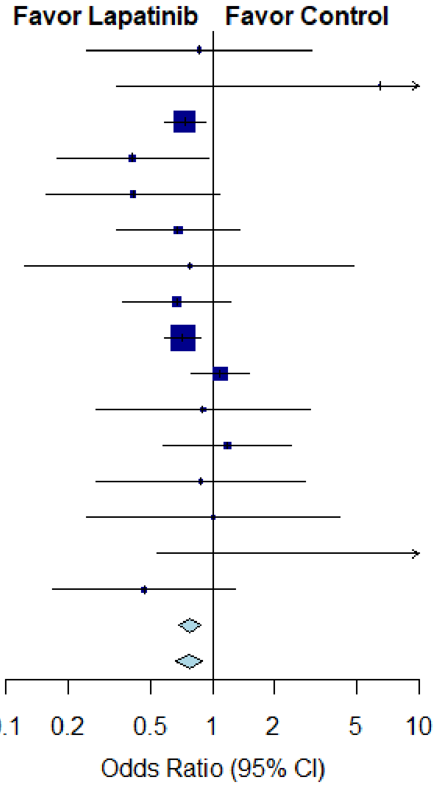

Figure S5-2. Effect of lapatinib on headache, when NCT00075270, NCT00486954, and NCT00281658 were excluded.

Source	Lapatinib Events	Lapatinib Total	Control Events	Control Total	OR (95% CI)
NCT00490139	88	2061	123	2076	0.71 [0.53; 0.94]
NCT00073528	35	654	54	624	0.60 [0.38; 0.93]
NCT00429299	2	46	2	36	0.77 [0.10; 5.77]
NCT00390455	3	141	2	137	1.47 [0.24; 8.92]
NCT00524303	2	31	1	32	2.14 [0.18; 24.86]
NCT00968968	1	18	0	17	3.00 [0.11; 78.81]
NCT01160211	6	118	6	116	0.98 [0.31; 3.14]
Total (fixed effect)	137	3069	188	3038	0.71 [0.56; 0.89]
Total (random effects)					0.71 [0.56; 0.89]

Heterogeneity: χ^2_{6} = 3.05 (P = .80), I^2 = 0%

Test for overall effect (random effects): z = -3.01 (P = .0026)

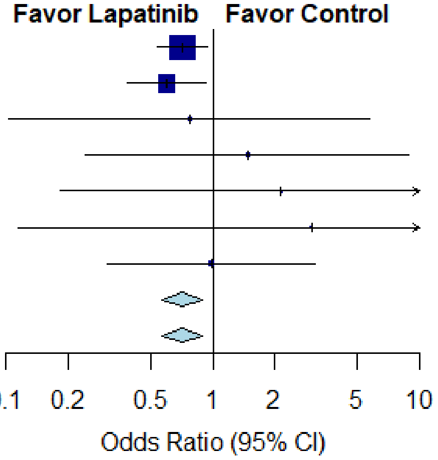

Figure S5-3. Effect of lapatinib on bone pain, when NCT00075270, NCT00486954, and NCT00281658 were excluded.

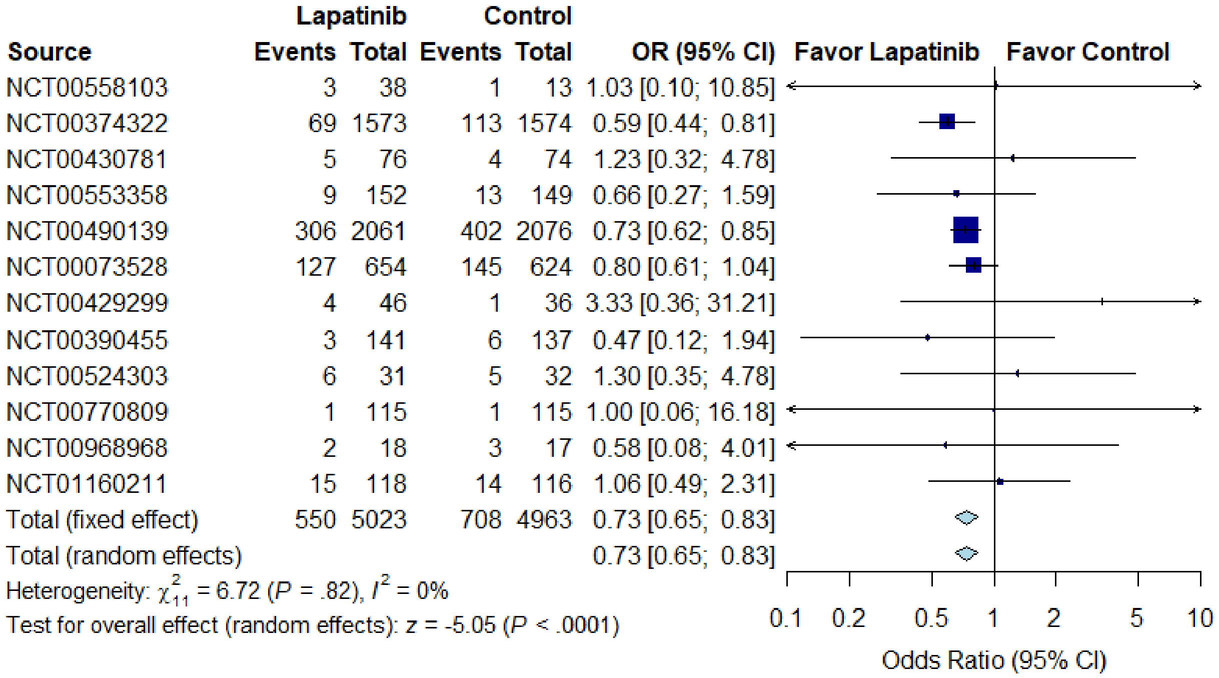

Figure S5-4. Effect of lapatinib on arthralgia, when NCT00075270, NCT00486954, and NCT00281658 were excluded.

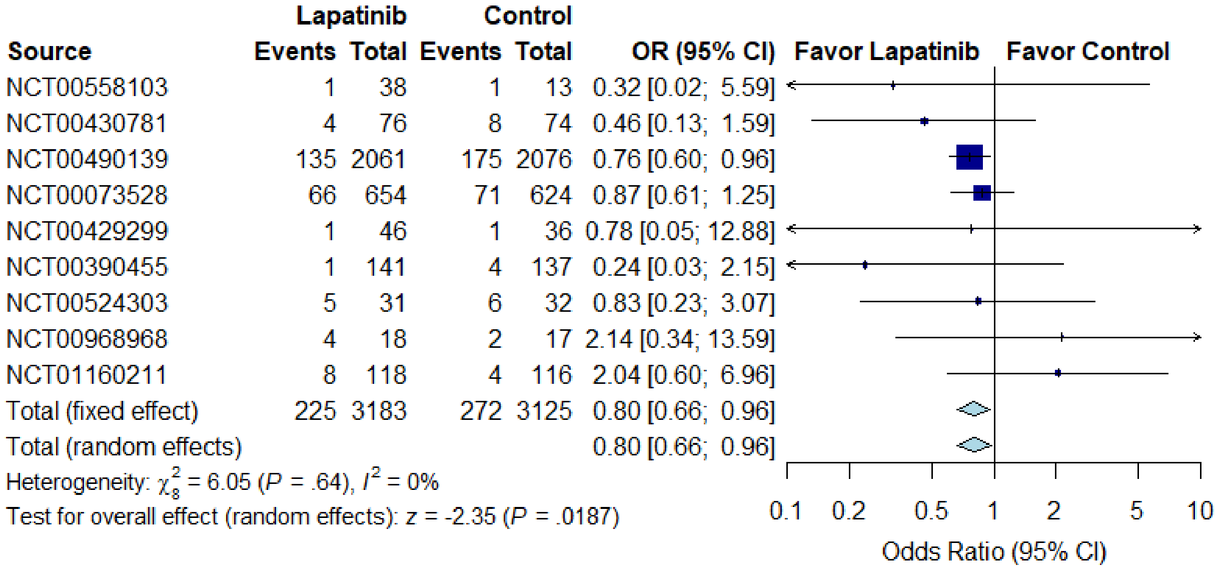

Figure S5-5. Effect of lapatinib on pain in extremity, when NCT00075270, NCT00486954, and NCT00281658 were excluded.

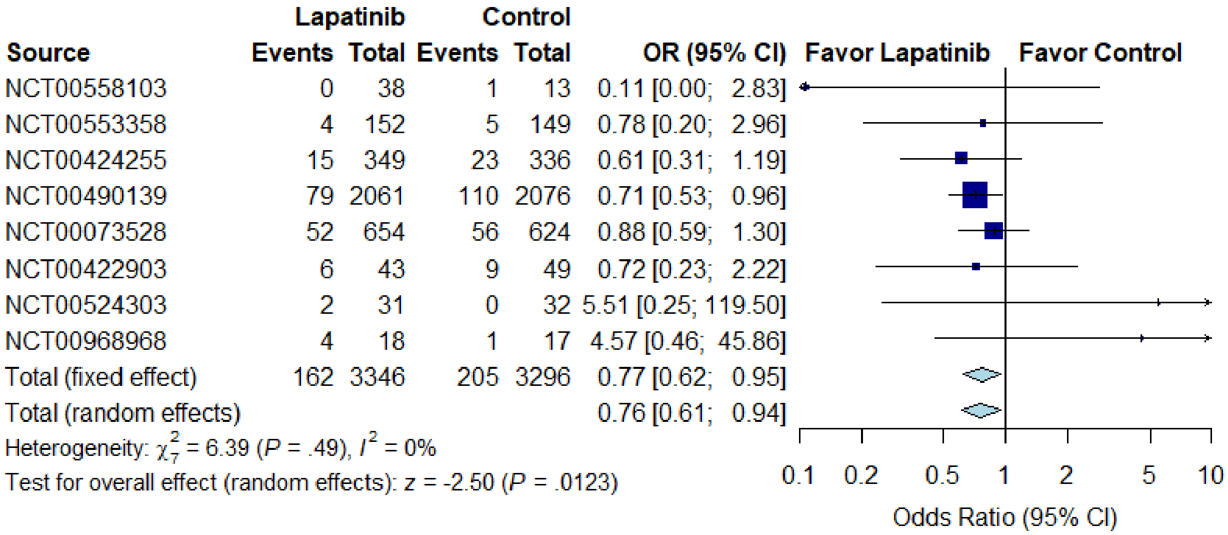

Figure S5-6. Effect of lapatinib on musculoskeletal pain, when NCT00075270, NCT00486954, and NCT00281658 were excluded.

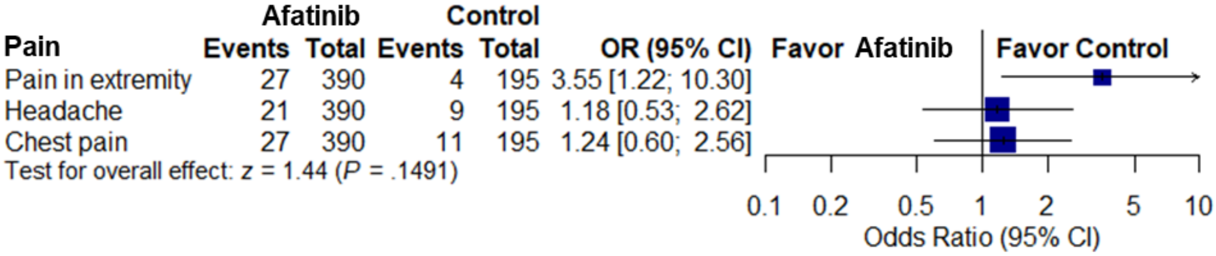

Figure S6. Effects of afatinib on various forms of pain. Data from NCT00656136.[1]

Formula for Calculation of Odds Ratio (OR):

	Diseased	Healthy
Exposed	D_E	H_E
Not exposed	D_N	H_N

OR = $(D_E/H_E)/(D_N/H_N) = (D_E H_N)/(D_N H_E)$

For synthesis of the data from multiple trials, the detailed procedure of the random-effects model[2] used to calculate the weight of each trial used in the meta-analysis can be found in reference.[3]

References

1 Miller, V. A. *et al.* Afatinib versus placebo for patients with advanced, metastatic non-small-cell lung cancer after failure of erlotinib, gefitinib, or both, and one or two lines of chemotherapy (LUX-Lung 1): a phase 2b/3 randomised trial. **13**, 528-538 (2012).

2 Veroniki, A. A. *et al.* Methods to estimate the between-study variance and its uncertainty in meta-analysis. *Res Synth Methods* **7**, 55-79, doi:10.1002/jrsm.1164 (2016).

3 Schwarzer, G., Carpenter, J. R. & Rücker, G. *Meta-analysis with R*. (Springer, 2015).

ROBIS analysis

Phase I

Category	**Target question (e.g. overview or guideline)**
Patients/Population(s):	Cancer patients
Intervention(s):	Therapy with lapatinib
Comparator(s):	Same therapy to the intervention group except lapatinib
Outcome(s):	Number of patients complaining about pains as adverse effects

Phase II

1. Study eligibility criteria

Signaling questions/Reviews	1.1 Did the review adhere to predefined objectives and eligibility criteria?	1.2 Were the eligibility criteria appropriate for the review question?	1.3 Were eligibility criteria unambiguous?	1.4 Were all restrictions in eligibility criteria based on study characteristics appropriate	1.5 Were any restrictions in eligibility criteria based on sources of information appropriate
Bone pain	Y	Y	Y	Y	Y
Arthralgia	Y	Y	Y	Y	Y
Musculoskeletal pain	Y	Y	Y	Y	Y
Headache	Y	Y	Y	Y	Y
Pain in extremity	Y	Y	Y	Y	Y
Myalgia	Y	Y	Y	Y	Y
Overall	Y	Y	Y	Y	Y

2. Identification and selection of studies

Signaling questions/Reviews	2.1 Did the search include an appropriate range of database/electronic sources for published and unpublished reports?	2.2 Were methods additional to database searching used to identify relevant reports?	2.3 Were the terms and structure of the search strategy likely to retrieve as many as eligible studies as possible	2.4 Were restrictions based on date, publication format, or language appropriate?	2.5 Were efforts made to minimize error in selection of studies
Bone pain	Y	N	Y	Y	Y

ROBIS analysis

Arthralgia	Y	N	Y	Y	Y
Musculoskeletal pain	Y	N	Y	Y	Y
Headache	Y	N	Y	Y	Y
Pain in extremity	Y	N	Y	Y	Y
Myalgia	Y	N	Y	Y	Y
Overall	Y	N	Y	Y	Y

3. Data collection and study appraisal

Signaling questions/Reviews	3.1 Were efforts made to minimize error in data collection?	3.2 Were sufficient study characteristics available for both authors and readers to be able to interpret the results?	3.3 Were all relevant study results collected for use in the synthesis?	3.4 Was risk of bias (or methodologic quality) formally assessed using appropriate criteria?	3.5 Were efforts made to minimize error in risk of bias assessment?
Bone pain	Y	Y	Y	Y	Y
Arthralgia	Y	Y	Y	Y	Y
Musculoskeletal pain	Y	Y	Y	Y	Y
Headache	Y	Y	Y	Y	Y
Pain in extremity	Y	Y	Y	Y	Y
Myalgia	Y	Y	Y	Y	Y
Overall	Y	Y	Y	Y	Y

4. Synthesis and findings

Signaling questions/Reviews	4.1 Did the synthesis include all studies that it should?	4.2 Were all predefined analyses reported or departures explained?	4.3 Was the synthesis appropriate given the nature and similarity in the research question, study designs, and outcomes across included studies?	4.4 Was between-study variation minimal or addressed in the synthesis	4.5 Were the findings robust, for example, as demonstrated through funnel plot or sensitivity analyses?	4.6 Were biases in primary studies minimal or addressed in the synthesis?
Bone pain	Y	Y	Y	Y	Y	Y

ROBIS analysis

Arthralgia	Y	Y	Y	Y	Y	Y
Musculoskeletal pain	Y	Y	Y	Y	Y	Y
Headache	Y	Y	Y	Y	Y	Y
Pain in extremity	Y	Y	Y	Y	Y	Y
Myalgia	Y	Y	Y	Y	Y	Y
Overall	Y	Y	Y	Y	Y	Y

Phase III

Risk of bias in the review

Signaling questions/Reviews	A. Did the interpretation of findings address all of the concerns identified in domain 1 to 4 of Phase 2?	B. Was the relevance of identified studies to the review's research question appropriately considered?	C. Did the reviewers avoid emphasizing results on the basis of their statistical significance?
Bone pain	Y	Y	N
Arthralgia	Y	Y	N
Musculoskeletal pain	Y	Y	N
Headache	Y	Y	N
Pain in extremity	Y	Y	N
Myalgia	Y	Y	N
Overall	Y	Y	N
